# Disseminated Kaposi sarcoma patients exhibit an expanded population of CD8+CD57+ T cells and an immunosenescence profile

**DOI:** 10.3389/fimmu.2025.1625386

**Published:** 2025-10-24

**Authors:** Julio Flores-Gonzalez, Lucero A. Ramón-Luing, Beda Islas-Muñoz, Patricia Volkow-Fernández, Leslie Chavez-Galan

**Affiliations:** ^1^ Laboratory of Integrative Immunology, Instituto Nacional de Enfermedades Respiratorias Ismael Cosio Villegas, Mexico, Mexico; ^2^ Infectious Diseases Department, Instituto Nacional de Cancerología, Mexico, Mexico

**Keywords:** human herpesvirus-8/Kaposi sarcoma herpesvirus, CD8+CD57+ T-cells, immunosenescence, flow cytometry, valganciclovir

## Abstract

**Introduction:**

Kaposi’s sarcoma herpesvirus (KSHV) remains the most common opportunistic malignancy that contributes to morbidity and mortality among persons living with HIV (PLWH) worldwide. The immune response in PLWH can exhibit signs of functional exhaustion, characterized by CD57 expression and mitochondrial dysfunction in T-cells. Valganciclovir (VGC), as an add-on therapy in patients with disseminated Kaposi Sarcoma/human immunodeficiency virus (DKS/HIV), modulates the activation of T-cell subsets; however, its effect on the T-cell immunosenescence profile is unclear.

**Methods:**

This study evaluated the T-cell immunosenescence profile in DKS/HIV patients who received two treatment schedules: A group received antiretroviral therapy (cART) as conventional therapy (CT, n=10), while a second group received an experimental regimen, consisting of VGC initially plus cART (VGC+cART, n=10) by the fourth week. Mononuclear cells from DKS/HIV patients were obtained at baseline (W_0_) and at weeks W_4_ and W_12_ of treatment. T-cells were labeled with cell markers such as CD3, CD4, CD8, CD27, CD57, KLRG1, PD-1, TIM-3, and GLUT1, as well as soluble molecules and a proteome profile array of proteins related to proteases.

**Results:**

Data showed that DKS/HIV patients have an increased frequency of GLUT1+ T-cells at diagnosis, which was not modified after treatment initiation. The presence of CD8+CD57+KLRG1+ T-cells was expanded in DKS/HIV patients and maintained across follow-up once VGC+cART treatment was started. Although DKS/HIV patients display high plasma levels of soluble ligands for KLRG1 (E-cadherin) and TIM-3 (Gal-9) at diagnosis, together with proteases associated with the regulation of T-cells and the induction of T-cell immunosenescence, both treatment schedules reduce their soluble levels after 12 weeks of follow-up.

**Discussion:**

The microenvironment generated in DKS/HIV patients increases the frequency of T-cells exhibiting an immunosenescence phenotype, and this effect is independent of the treatment schedule used, suggesting that during coinfection, a chronic immunosuppressive microenvironment may develop, impairing immune surveillance and resilience. These results could be explored to identify novel therapeutic approaches.

## 1 Introduction

Kaposi’s sarcoma herpesvirus (KSHV), also known as human herpesvirus 8 (HHV-8), is more prevalent among men who have sex with men (MSM), particularly those at high risk for human immunodeficiency virus (HIV) infection and other sexually transmitted diseases (1). Although the widespread use of combined antiretroviral therapy (cART) has significantly reduced the incidence of disseminated Kaposi’s sarcoma (DKS) in people living with HIV (PLWH), DKS remains the most common HIV-associated malignancy worldwide and continues to contribute to significant morbidity and mortality (2). Valganciclovir (VGC) is an add-on therapy for HHV-8 to reduce the viral replication and decrease mortality attributable to severe immune reconstitution inflammatory syndrome (IRIS) episodes (3). VGC is an oral prodrug of ganciclovir that inhibits viral DNA synthesis and is a standard gold treatment for cytomegalovirus end-organ disease and secondary prophylaxis. Clinical trials have proposed the clinical use of VGC as an antiviral to reduce HHV-8 replication in KS/HIV patients (4, 5).

The HHV-8/HIV coinfection leads to chronic immune activation and persistent inflammation, but the immunopathological mechanisms that drive disease progression in DKS/HIV patients remain incompletely understood. Immunosenescence is a process that has been implicated in immune dysfunction during chronic viral infections. As a consequence, the immune cells display characteristics such as impaired proliferation, altered surface marker expression, metabolic dysregulation, and reduced effector function (6).

In T-cells, immunosenescence is characterized by increased glycolytic activity, which is linked to the upregulation of glucose transporters (GLUTs), such as GLUT1. This metabolic shift is associated with enhanced mitochondrial biogenesis and altered oxidative phosphorylation (7). In the context of HIV, chronic infection induces GLUT1 overexpression on T-cells, contributing to persistent immune activation and eventual cellular exhaustion (8, 9). In addition, factors such as comorbidities, immunodeficiency, and HIV-related immune remodeling may accelerate immunosenescence in PLWH (10).

Phenotypically, senescent T-cells are often characterized by the loss of the costimulatory molecule CD27 and the expression of inhibitory or senescence-associated markers such as CD57 and KLRG-1 (killer cell lectin-like receptor G1) (11, 12). The presence of these markers reflects chronic antigenic stimulation and correlates with impaired proliferative capacity and reduced responsiveness to new antigens. Notably, reports have identified immunosenescent T-cell profiles, along with mitochondrial dysfunction, in patients with KS and HIV coinfection (13, 14).

Previous studies have reported that DKS/HIV patients exhibit additional immune alterations, including CD8+ T cells with high CD57 expression and impaired mitochondrial activity (14), and natural killer (NK) cells with elevated PD-1 expression, features that are not fully reversed by cART or cART combined with valganciclovir (VGC+ART) treatment. However, the use of VGC contributes to improving immune reconstitution during the first weeks of treatment (15, 16). Another report suggested that persistent immune dysregulation may contribute to the pathogenesis of DKS despite virologic control, potentially leading to irreversible immune exhaustion and senescence (17).

Based on this evidence, we hypothesized that DKS/HIV patients exhibit distinct immunosenescence signatures in their T cell populations and plasma. This study aimed to characterize immunosenescence and immune checkpoint expression profiles in T cells from DKS/HIV patients undergoing either conventional cART treatment (CT) or an experimental regimen, which included initial VGC and, after four weeks, initiation of cART (VGC+cART). Finally, also investigated the relationship between these cellular markers and soluble immune mediators associated with immunosenescence.

## 2 Materials and methods

### 2.1 Study population

The study was approved by the Ethics Committee of the Instituto Nacional de Enfermedades Respiratorias (B30–20) and the Instituto Nacional de Cancerología (015/031/INI) (CEI/950/15), registered at NIH Clinical Trials (ID NCT03296553). All procedures were conducted in accordance with the 1964 Helsinki Declaration and the ethical standards of the Institutional Ethics Committees.

A total of 40 participants were included for this study, all men who have sex with men (MSM) > aged 18 years old, into four groups (**Supplementary Figure S1**):

1. Ten MSM that tested negative for HIV during screening (hereafter called HIV-)
2. Ten MSM who tested HIV positive during the screen were asymptomatic with >350 CD4+ cells/mL (hereafter called HIV+)
3. Ten DKS/HIV patients that participated in the randomized clinical trial (RCT) in the control group and received cART according to the current Mexican Guidelines, hereafter called conventional treatment (CT).
4. Ten DKS/HIV patients participated in the RCT in the experimental group, received VGC 900 mg BID at week 0 (W_0_); subsequently, cART was initiated at week (W_4_) and continued with both drugs until W_48_, hereafter called VGC+cART.

Control groups (1 and 2) were recruited at Clinica Especializada Condesa, the Mexico City specialized clinic for HIV diagnosis and care, and participants were paired by age with DKS/HIV patients. In contrast, DKS/HIV patients (groups 3 and 4) were enrolled during 2015–2018 as part of the RCT and followed at the Instituto Nacional de Cancerología, Mexico City. DKS was defined as the presence of KS pulmonary disease and/or ≥30 KS skin lesions, with or without lymphedema, and/or lymph node involvement, and/or GIT KS involvement (biopsy-proven at least in one site).

All HIV+ participants, group 2, 3 (CT), and 4 (VGC+cART) patients were cART naïve at enrollment time. The VGC regimen used for VGC+cART was based on the clinical trial’s aim (ID NCT03296553) to reduce the risk of severe IRIS-KS (Immune Reconstitution Inflammatory Syndrome) development.

Severe-IRIS-KS was defined as an abrupt clinical deterioration after cART initiation alongside the presence of at least two clinical and at least three laboratory criteria:

Clinical criteria: 1) Fever (without identified concomitant infection), 2) increase in the size or number of KS lesions, 3) exacerbation of lymphedema, 4) appearance or increase of otherwise unexplained lung opacities on the chest images with a negative Gallium-Scan, and 5) appearance or increase of volume of pleural effusion. Laboratory criteria: 1) Thrombocytopenia <100,000 platelets/ml; 2) Anemia (decrease of at least 1 g/dl from previous measure with no obvious bleeding), 3) Hyponatremia <135 mEq/L, and 4) Hypoalbuminemia <3.5 g/dL (3).

The chemotherapy regimen used was vincristine 2 mg (reduced to 1 mg if albumin was <3 g/dL) plus bleomycin 15 UI, which was indicated by the treating physician either at the time of diagnosis (when the participant had extensive KS involvement or bulky oropharyngeal lesions before cART), or at follow-up in case of S-IRIS-KS or persistence of extensive KS lesions. There were no differences in the number of patients receiving vincristine/bleomycin or the number of cycles administered between the CT and the VGC+cART.

Clinical and demographic information for DKS/HIV participants was collected at enrollment and during the follow-up visits of the study; complete information is available in the RCT report (3, 18). For this study, blood samples were collected at the W_0_, W_4_, and week 12 (W_12_) to obtain peripheral blood mononuclear cells (PBMCs) of DKS/HIV patients; an additional plasma sample was collected at weeks (W_0_, W_2_, W_4_, W_8_, W_12_, W_16_, and W_24_). However, since the samples were residual material from clinical laboratory tests and the EDTA tubes used had a limited volume capacity, it was not possible to obtain PBMCs from every follow-up visit.

### 2.2 Sample processing

A venous blood sample was collected into a BD Vacutainer^®^ EDTA tube, and PBMCs were separated by density gradient centrifugation by standard LymphoprepTM (Accurate Chemical-Scientific, Westbury, NY, USA) and cryopreserved until use. Plasma samples were separated and stored at −70 °C until they were used. After thawing the samples, the viability of the cells was determined by trypan blue exclusion assay; a minimum of 70% viability was required to process the cells.

### 2.3 Immunophenotyping using flow cytometry

PBMCs were prepared to evaluate cell surface marker expressions using monoclonal antibodies (mAbs) to CD3, CD4, CD8, CD27, CD57, KLRG1, PD-1, TIM-3, and GLUT1. Fluorescence Minus One condition was stained and acquired in parallel to identify background levels of staining; dead cells were excluded using viability staining Zombie Red Dye solution (**Supplementary Table S1**). The data were acquired using a FACS Aria II flow cytometer (BD Biosciences, San Jose, CA, USA) equipped with the FACSDiva 6.1.3 software. In each condition, at least 50,000 events of interest were acquired per sample. The flow cytometry data file (FCS) was analyzed using Flow Jo (Flow Jo, LLC, Ashland, OR, USA) ™ v10.9.0. A representative flow cytometry set-up gate strategy is shown in **Supplementary Figure S2**.

### 2.4 Enzyme-linked immunosorbent assay

Soluble levels of TIM3 and Gal-9 were measured in the plasma by standard enzyme-linked immunosorbent assay (ELISA) following the manufacturer’s recommendations (**Supplementary Table S1**). The optical density at 450 nm was measured using a microplate reader spectrophotometer (Imark, Bio-Rad, Hercules, CA, USA. All measurements were quantified by interpolation with the corresponding standard curve.

### 2.5 Flow cytometry-based multiplex immunoassays

Cytokines and cytotoxic molecules were measured in plasma using a human CD8/NK panel (13 plex) provided by BioLegend (**Supplementary Table S1**). This panel allows the quantification of L-17A, IL-2, IL-4, IL-10, IL-6, TNF-α, Fas, FasL, IFN-γ, Granzyme A, Granzyme B, Perforin, and Granulysin. The bead assays were conducted strictly following the manufacturer’s guidelines. Data were acquired using flow cytometry (FACS Aria II, Becton Dickinson) and analyzed with the LEGENDplex™ Data Analysis Software Suite.

### 2.6 Proteome profile array of protease-related proteins

Thirty-five protease-related proteins in the plasma from KS/HIV were evaluated with the Proteome Profiler™ kit (R&D Systems Inc. Catalog ARY021B); four biological replicates were included for either CT or VGC+cART. The selected patients were matched by age to avoid misinterpretation of molecule levels. Patients were randomly selected from each group, ensuring they were representative in terms of cytokine levels and phenotypical characterization results. The total plasma protein was quantified using a Qubit™ assay kit and the Qubit 2.0 Fluorometer (Life Technologies, Waltham, MA, USA), and the screenings were performed according to the manufacturer’s instructions. The Proteome Profiler™ kit was evaluated in duplicate to analyze the relative level of target proteins. Briefly, 200 μg of total protein was diluted in a final volume of 1.5 mL of array buffer, added to the nitrocellulose membranes, and incubated at 4°C with gentle shaking overnight. Then, the membranes were washed to remove unbound proteins, and subsequently, a cocktail of biotinylated detection antibodies was added. Finally, streptavidin-HRP and chemiluminescent detection reagents were added to detect protein spots, and the Imaging System from Bio-Rad (ChemiDocTM XRS+System) was used to visualize them.

Pixel density was determined by NIH-provided online ImageJ software (Version 2.1.0/1.53c), and density values were normalized to the reference spots in the Proteome Profiler™ kit.

### 2.7 Statistical analysis

The normality of the data was tested using the D’Agostino–Pearson test. Non-normally distributed variables are shown as median value and interquartile range (IQR, 25–75). The Mann–Whitney U test was used to compare two groups, while the Kruskal–Wallis test, followed by Dunn’s *post-hoc* test, was used for multiple comparisons. Data are shown as median values and interquartile ranges (IQR, 25–75). In all tests, the standard of significance was set at p < 0.05. Data were analyzed using GraphPad Software (v 9.0.1).

The Spearman correlation matrix was produced in R version 4.2.0 using the corrplot version 0.92 package. The color and size of the circles in the diagram represent the magnitude and direction of the correlation. The p-values were also calculated, and the data points with p-values greater than 0.05 are not shown in the diagram, indicating a lack of statistical confidence.

## 3 Results

### 3.1 Description of the study population

Demographic data of DKS/HIV patients divided into CT and VGC+cART, along with data from control groups (both HIV-positive and HIV-negative), have been reported previously (18). Here, descriptive data of the twenty DKS/HIV patients included in this study are shown in **Supplementary Table S2**. The median age of patients was 33 years (range, 26-41), and eleven (55%) developed IRIS at various time points: one at W_2_, seven at W_4_, two at W_6_, and one at W_9_. Half of DKS/HIV patients showed opportunistic infections (OIs); three (15%) had syphilis, three (15%) had disseminated *Mycobacterium avium complex* (MAC) (15%), one (5%) had histoplasmosis, and three (15%) were positive for *Helicobacter pylori*.

Concerning HIV-1 viral load (VL), before the initiation of cART treatment, levels were elevated and were reduced significantly by W_4_ (p=0.0004). In contrast, the high HHV-8 VL gradually decreased, maintaining statistical significance until W_24_ (p=0.0087). Patients diagnosed with DKS/HIV presented a low absolute CD4+ T-cell count at Week 0, approximately half of the mean observed at W_24_. Additionally, the CD8+ T lymphocyte count was inversely related, resulting in a CD4/CD8 ratio consistent with expectations throughout the follow-up period.

### 3.2 GLUT1+ T-cells are increased in DKS/HIV

**Figure 1:**
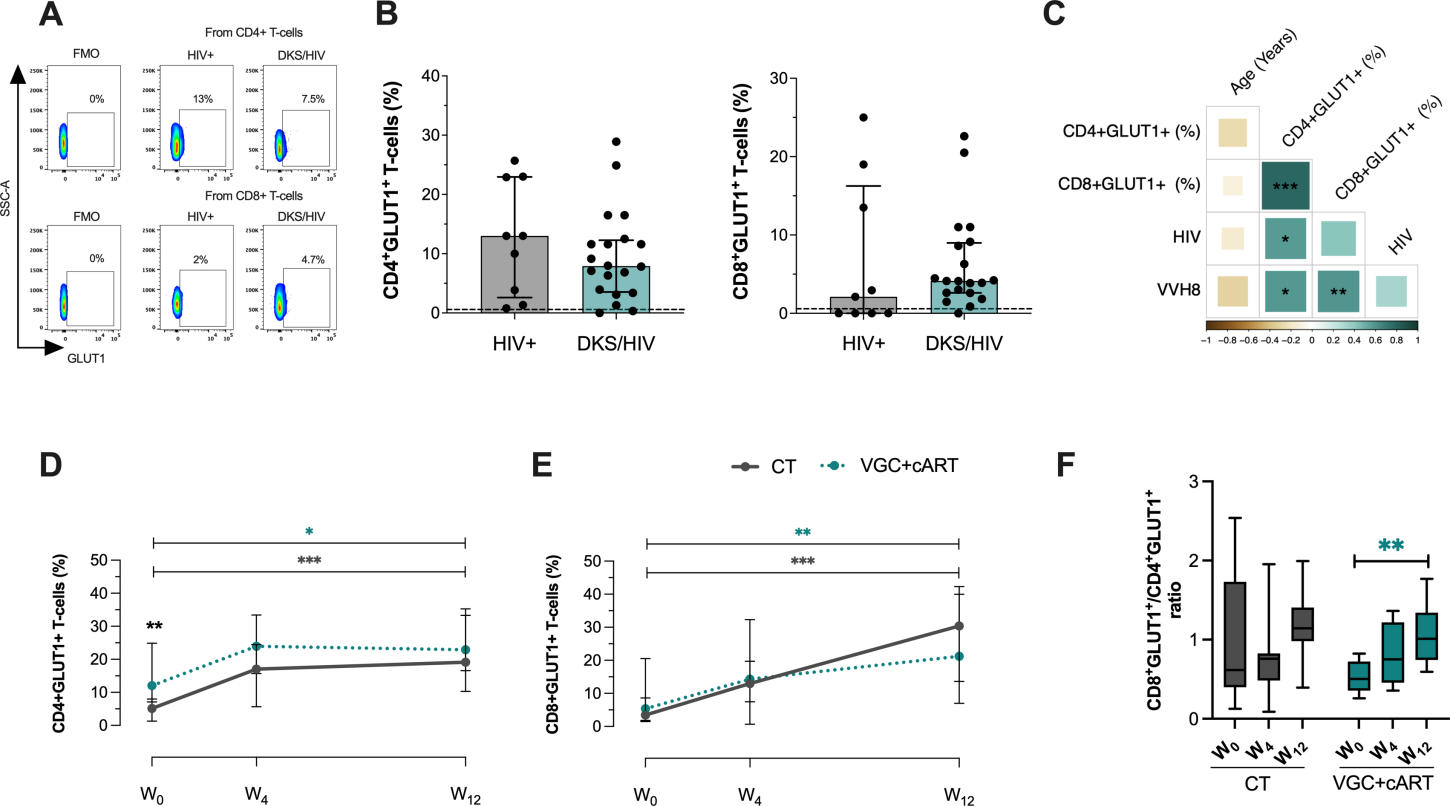
DKS/HIV patients exhibit an increased frequency of CD4+GLUT1+ and CD8+GLUT1+ T-cells. **(A)** Representative flow cytometry of the expression of GLUT-1 within CD4+ and CD8+ T-cells of HIV+ subjects and DKS/HIV patients. **(B)** The frequency of CD4+GLUT1+ and CD8+GLUT1+ T-cells was assessed in the study groups at the time of diagnosis. **(C)** Spearman correlation matrix between GLUT1+ T-cells, age, plasma IL-10 levels, and HIV/VHH8 viral load in DKS/HIV patients at diagnosis. **(D)** The frequency of CD4+GLUT1+ T-cells and **(E)** CD8+GLUT-1+ T-cells in the DKS/HIV patient groups across follow-ups is divided by treatment. **(F)** The proportion of CD4+GLUT1+ and CD8+GLUT1+ T-cells in the DKS/HIV patient groups during the follow-up, divided by treatment. Data are represented as median and IQR values. The dashed black line represents the median value for HIV-negative men. Statistical comparisons were performed by the Kruskal-Wallis test (green to VGC+cART and grey to CT group) and Mann–Whitney U test (black asterisk); * p < 0.05, ** p < 0.01, *** p < 0.001.

**Figure 1A** shows a representative flow cytometry dot plot of GLUT-1 expression within CD4+ and CD8+ T-cells evaluated in HIV+ subjects and DKS/HIV patients. Analysis revealed that the frequency of CD4+ and CD8+ T-cells positive for GLUT1 in DKS/HIV patients was similar to that of HIV+ at W_0_ (**Figure 1B**). Compared to HIV-negative individuals, HIV+ subjects had a higher frequency of CD4+ GLUT1+ T-cells (p=0.0431). Similarly, DKS/HIV patients had an elevated frequency of CD4+ GLUT1+ (p=0.0203) and CD8+ GLUT1+ T-cells (p=0.0412) (**Supplementary Table S3**).

A positive correlation was observed between the frequency of CD4+ GLUT1+ T-cells and HIV-1 viral load (rs=0.60; p=0.0119) and HHV-8 viral load (rs=0.60; p=0.0113), while the frequency of CD8+ GLUT1+ T-cells showed a positive correlation only with HHV-8 viral load (rs=0.60; p=0.0092). This data suggests that HHV-8 could be associated with a significant increase of GLUT1 on CD8+ T-cells in DKS/HIV patients (**Figure 1C** and **Supplementary Figure S3A**).

Previously, we reported that DKS/HIV recovered the CD4+ T-cell count across follow-up, mainly with VGC+cART therapy (18). To investigate whether VGC+cART treatment would reduce the presence of GLUT1+ T-cells, GLUT1 expression was analyzed on T-cells at W_0_, W_4_, and W_12_ in DKS/HIV patients (**Supplementary Figure S4A**). At W_0_, patients who received the VGC+cART showed a higher frequency of CD4+GLUT1+ T-cells than those who received CT (p=0.0037); however, at W_12_, both groups showed a twofold increase compared to W_0_ (CT, p=0.0001; VGC+cART, p=0.0138) (**Figure 1D**). The frequency of CD8+GLUT1+ T-cells was not different at W_0_. However, at W_12_, both groups increased their frequency twice compared to W_0_ (CT, p=0.0001; VGC+cART, p=0.0013) (**Figure 1E**). In addition, the ratio between the two subpopulations increased proportionally over the follow-up period (**Figure 1F**).

These results suggest an increase in glucose metabolism in CD4+T cells at W_0_ and in both CD4+T and CD8+ T cells at W_12_. To exclude the possibility that this increase resulted from chemotherapy interactions (bleomycin and vincristine), we reanalyzed the data considering chemotherapy as a dependent variable. DKS/HIV were divided into those who received chemotherapy and those who did not. The data confirmed that the findings were independent of the chemotherapy (**Supplementary Figure S4B**).

### 3.3 VGC+cART scheme induced an expansion of CD8+CD57+ T-cells in DKS/HIV patients

**Figure 2:**
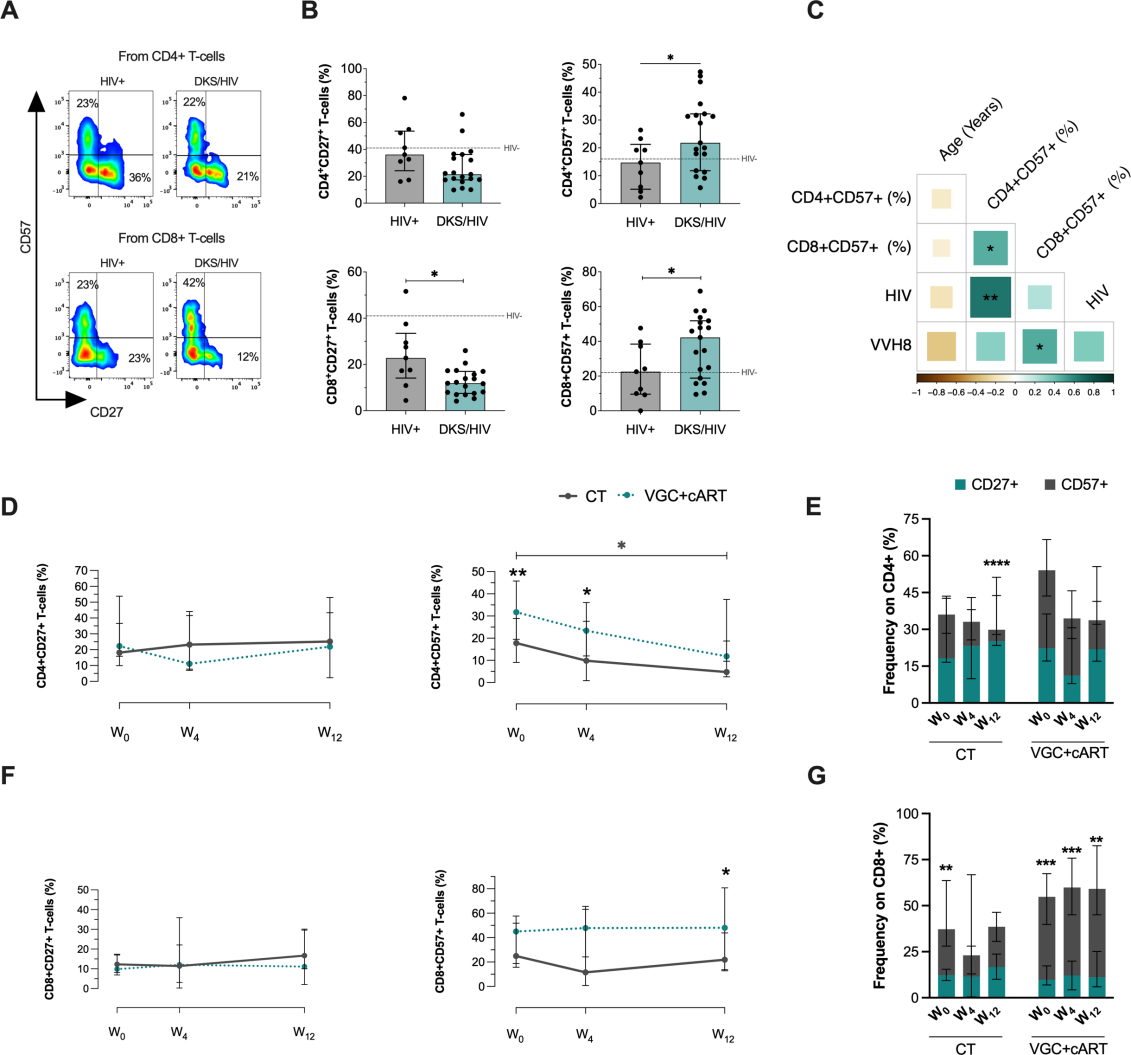
T-cells from DKS/HIV patients show a distinct CD57 and CD27 expression profile. **(A)** Representative flow cytometry of the expression of CD27 and CD57 within CD4+ and CD8+ T-cells of HIV+ and DKS/HIV patients. **(B)** The frequency of CD27 and CD57 expression on T-cells was assessed in the study groups at the time of diagnosis. **(C)** Spearman correlation matrix between CD27+ and CD57+ T-cells, age, plasma IL-10 levels, and HIV/VHH8 viral load in DKS/HIV patients at diagnosis. **(D)** Frequency of CD4+CD27+ T-cells and CD4+CD57+ T-cells in the DKS/HIV patient groups across follow-up. **(E)** The proportion of CD27 and CD57 in CD4+ T-cells in the DKS/HIV patient groups during the follow-up, divided by treatment. **(F)** Frequency of CD8+CD27+ T-cells and CD8+CD57+ T-cells in the DKS/HIV patient groups across follow-up. **(G)** The proportion of CD27 and CD57 in CD8+ T-cells in the DKS/HIV patient groups during the follow-up, divided by treatment. Data are represented as median and IQR values. The dashed black line represents the median value for HIV-negative men. Statistical comparisons were performed by the Kruskal-Wallis test (green to VGC+cART and grey to CT group) and Mann–Whitney U test (black asterisk); * p < 0.05, ** p < 0.01, *** p < 0.001.

The increase in glucose metabolic activity in DKS/HIV patients could be related to T-cell activation and depletion status, similar to that of HIV patients (19). To corroborate, the expression of CD27 and CD57 was evaluated on T-cells to determine whether these cells from DKS/HIV patients exhibit an active or immunosenescence status (**Figure 2A**).

DKS/HIV patients had decreased the frequency of CD8+ T-cells compared to HIV+, specifically in CD27+ (p=0.0122) and an increase in CD57+ (p=0.0361) at W_0_. For CD4+ T-cells, only CD57+ cells increased at W_0_ (p=0.0358), while CD27+ cells remained unchanged (**Figure 2B**). Additional comparisons with HIV- are provided in **Supplementary Table S3**. The frequency of CD4+CD57+ T-cells positively correlated with HIV VL (rs=0.66; p=0.0017) (**Figure 2C**, **Supplementary Figure S3B**).

To confirm whether the increased expression of CD57 and CD27 was maintained after treatment, these markers were evaluated across the follow-up period (**Supplementary Figure S5A**). CD4+CD27+ T-cells did not show changes across the follow-up period, regardless of the treatment regimen. However, patients receiving the VGC+cART had a higher frequency of CD4+CD57+ T-cells at baseline and week 4 (W_0_, p=0.0094; W_4_, p=0.0272). Notably, CT gradually reduced the frequency of CD4+CD57+ T-cells, with a statistically significant difference observed between W_0_ and W_12_ (p=0.0350); this profile was not observed with VGC+cART (**Figure 2D**). The proportion of CD4+ T-cells expressing CD27 and CD57 was altered at W_12_ using the CT scheme, characterized by a loss of CD57 expression (p=0.0001) (**Figure 2E**).

Regarding CD8+ T-cells, the frequency of CD8+CD27+ remained unchanged across follow-up independently of the scheme treatment, and CD57 expression was higher in VGC+cART than CT at W_12_ (p=0.0330) (**Figure 2F**). VGC+cART patients displayed an altered ratio of CD8+ T-cells expressing CD27 and CD57; this profile was characterized by a significant increase of CD8+CD57+ T-cells (**Figure 2G**). Additionally, like GLUT1, the expressions of CD27 and CD57 were not associated with chemotherapy (**Supplementary Figure S5B**).

### 3.4 Both treatment schemes modulate T-cell senescence-like in DKS/HIV

A senescent/exhausted phenotype, characterized by expression of KLRG1, PD-1, and TIM-3, is observed during chronic viral infection (20). In this study, their expression is evaluated individually within the T-cell subsets, given our available cytometer.

**Figure 3:**
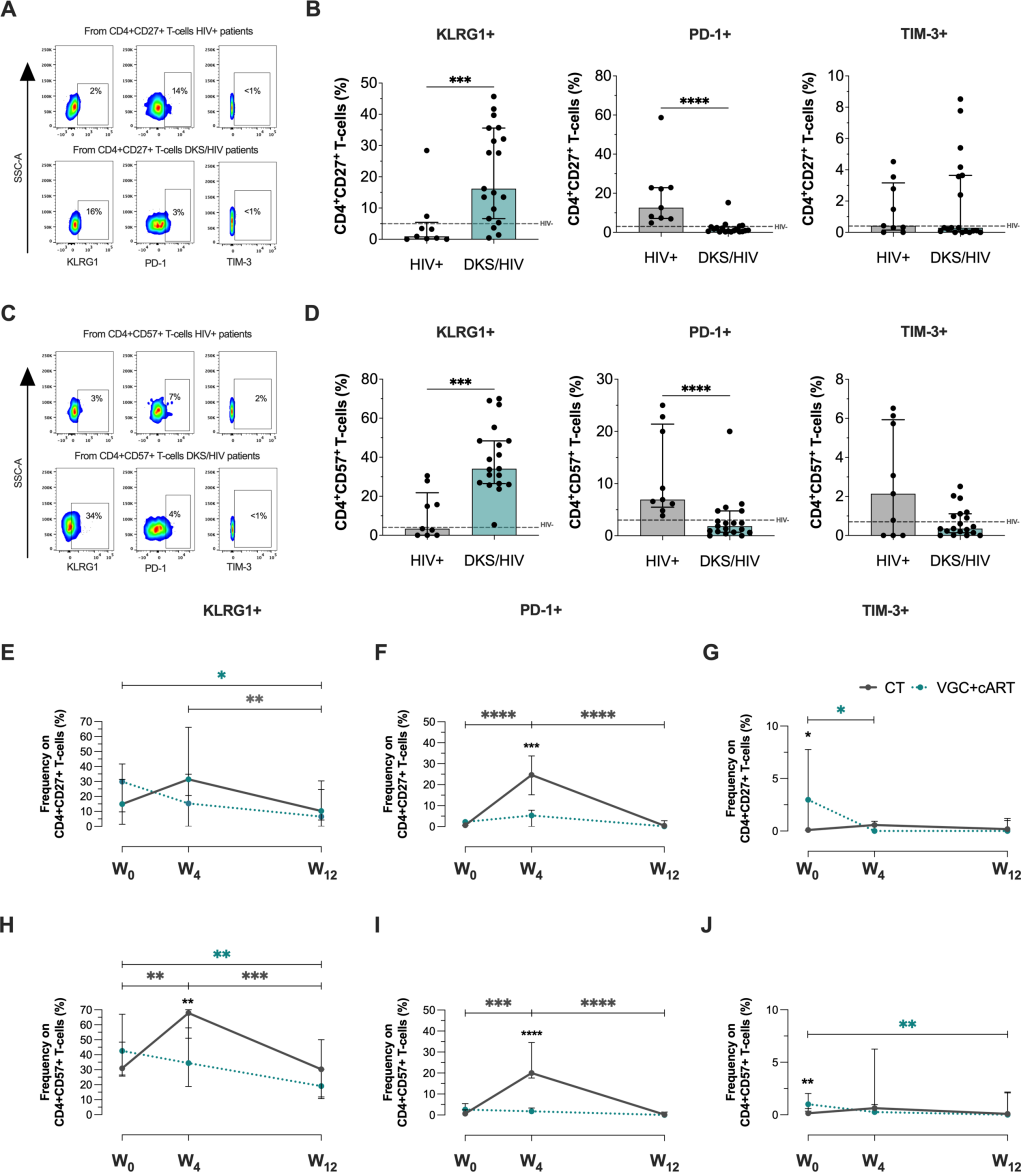
CD4+ T-cells in DKS/HIV patients display a senescence-associated phenotype characterized by high KLRG1 expression. **(A)** Representative flow cytometry of KLRG-1, PD-1, and TIM-3 expression within CD4+CD27+ T-cells of HIV+ and DKS/HIV patients. **(B)** Frequency of CD4+CD27+KLRG-1+, CD4+CD27+PD-1+, and CD4+CD27+TIM-3+ within HIV+ and DKS/HIV patients at diagnosis. **(C)** Representative flow cytometry of KLRG-1, PD-1, and TIM-3 expression within CD4+CD57+ T-cells of HIV+ and DKS/HIV patients. **(D)** Frequency of CD4+CD57+KLRG-1+, CD4+CD57+PD-1+, and CD4+CD57+TIM-3+ within HIV+ and DKS/HIV patients at diagnosis. **(E)** Frequency of KLRG-1, **(F)** PD-1, and **(G)** TIM-3 in CD4+CD27+ T-cells in the DKS/HIV patient groups across follow-up. **(H)** Frequency of KLRG-1, **(I)** PD-1, and **(J)** TIM-3 in CD4+CD57+ T-cells in the DKS/HIV patient groups during the follow-up. Data are represented as median and IQR values. The dashed black line represents the median value for HIV-negative men. Statistical comparisons were performed by the Kruskal-Wallis test (green to VGC+cART and grey to CT group) and Mann–Whitney U test (black asterisk); * p < 0.05, ** p < 0.01, *** p < 0.001.

To determine whether T-cells from DKS/HIV patients exhibit this phenotype, expression of these molecules was evaluated on CD4+ and CD8+ T-cells. CD4+CD27+ subpopulations were examined (**Figure 3A**). In comparison to HIV+, DKS/HIV showed an increase in the frequency of CD4+CD27+KLRG1+ (p=0.001) and a decrease in CD4+CD27+PD-1+ T-cells (p=0.0001) at W_0_, while TIM-3+ levels were similar between groups (**Figure 3B**). CD4+CD57+ subpopulations were tested (**Figure 3C**), compared to HIV+, DKS/HIV showed an increased frequency of CD4+CD57+KLRG1+ (p=0.001) and a decrease in CD4+CD27+PD-1+ T-cells (p=0.0001) at W_0_. TIM-3+ levels were similar between groups (**Figure 3D**). In each graphic, the frequency of HIV- is indicated as a dotted black line, and more details of comparisons, including HIV- groups, are shown in **Supplementary Table S3**.

Across follow-up, VGC+cART treatment decreased the frequency of CD4+CD27+KLRG1+ T-cells in DKS/HIV at W_12_ compared to W_0_ (p=0.0231), while CT decreased it at W_12_ compared to W_4_ (p=0.0051) (**Figure 3E**). VGC+cART did not affect the frequency of PD-1+, but CT induced a temporary increase at W_4_ compared to W_0_ (p=0.0001) and decreased again at W_12_ (p=0.0001) (**Figure 3F**). Patients treated with VGC+cART exhibited a higher frequency of CD4+CD27+TIM-3+ T-cells than those treated with CT (p=0.0361) at W_0_. However, this frequency decreased at W_4_ (p=0.0119) and remained at levels similar to those of CT thereafter (**Figure 3G**).

Otherwise, the VGC+cART decreased the frequency of CD4+CD57+KLGR1+ at W_12_ (p=0.0012), while CT increased this frequency at W_4_ compared to W_0_ (p=0.0053) and decreased it again at W_12_ (p=0.0005) (**Figure 3H**). The PD-1 expression on this cell subset of T-cells remained unaltered with VGC+cART; however, CT increased the frequencies of CD4+CD57+PD1+ at W_4_ (p=0.0004), but this decrease was observed again at W_12_ (p=0.0001) (**Figure 3I**). The frequency of CD4+CD57+TIM-3+ T-cells was higher in VGC+cART than in CT (p=0.0021) at W_0_; however, it decreased at W_12_ (p = 0.0099) to similar levels as those in CT (**Figure 3J**).

**Figure 4:**
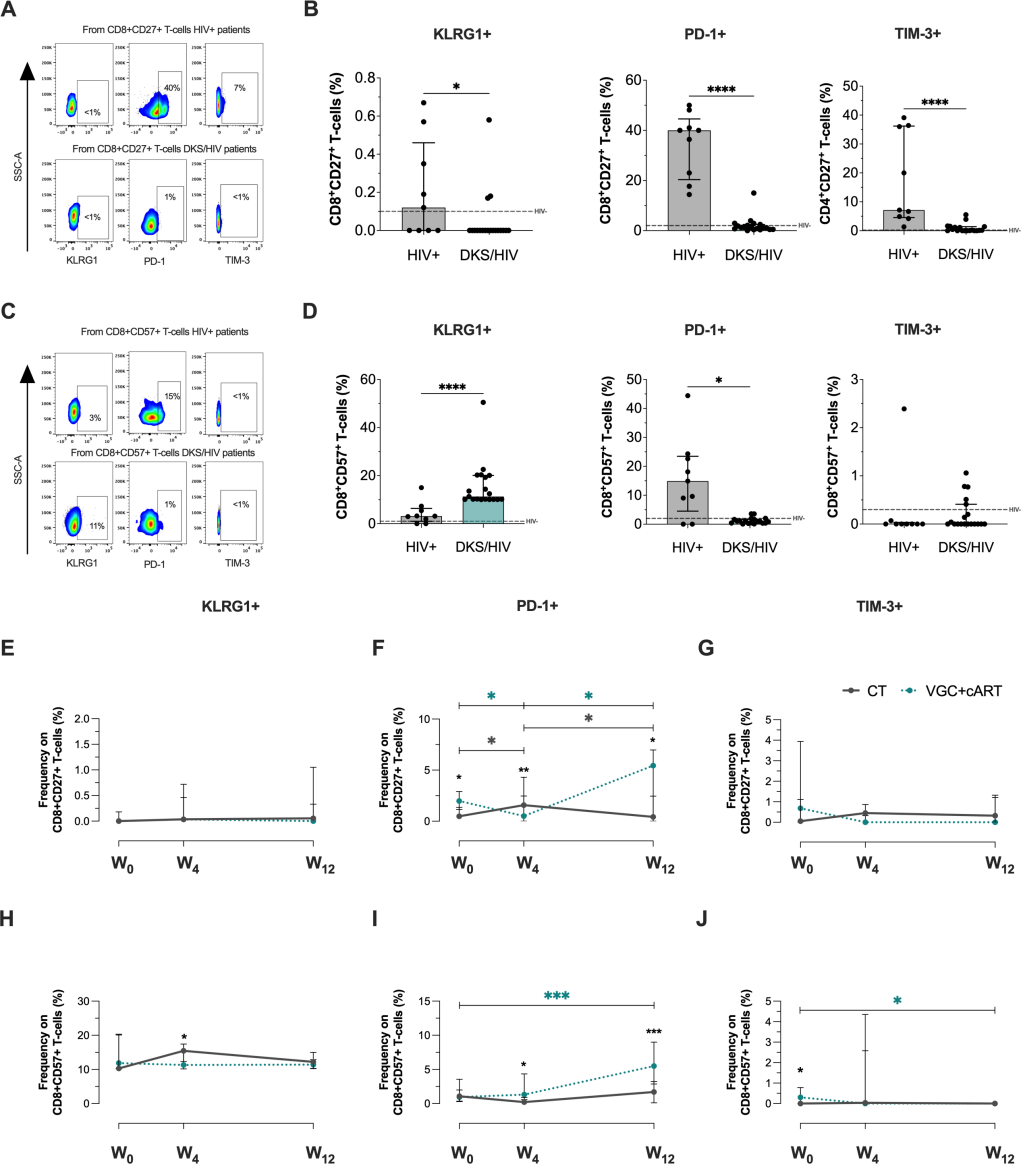
CD8+ T-cells in DKS/HIV patients are characterized by a CD8+CD57+KLRG1+ phenotype. **(A)** Representative flow cytometry of KLRG-1, PD-1, and TIM-3 expression within CD8+CD27+ T-cells of HIV+ and DKS/HIV patients. **(B)** Frequency of CD8+CD27+KLRG-1+, CD8+CD27+PD-1+, and CD8+CD27+TIM-3+ within HIV+ and DKS/HIV patients at diagnosis. **(C)** Representative flow cytometry of KLRG-1, PD-1, and TIM-3 expression within CD8+CD57+ T-cells of HIV+ and DKS/HIV patients. **(D)** Frequency of CD8+CD57+KLRG-1+, CD8+CD57+PD-1+, and CD8+CD57+TIM-3+ within HIV+ and DKS/HIV patients at diagnosis. **(E)** Frequency of KLRG-1, **(F)** PD-1, and **(G)** TIM-3 in CD8+CD27+ T-cells in the DKS/HIV patient groups across follow-up. **(H)** Frequency of KLRG-1, **(I)** PD-1, and **(J)** TIM-3 in CD8+CD57+ T-cells in the DKS/HIV patient groups during the follow-up. Data are represented as median and IQR values. The dashed black line represents the median value for HIV-negative men. Statistical comparisons were performed by the Kruskal-Wallis test (green to VGC+cART and grey to CT group) and Mann–Whitney U test (black asterisk); * p < 0.05, ** p < 0.01, *** p < 0.001.

Concerning CD8+CD27+ T-cells at W_0_ (**Figure 4A**), DKS/HIV patients showed a lower frequency of CD8+CD27+ T-cells positive to KLRG1, PD-1, and TIM-3 than HIV+ subjects (p<0.05, p<0.0001, p<0.0001, respectively) (**Figure 4B**). In the CD8+CD57+ T-cell subsets (**Figure 4C**), data showed that DKS/HIV has increased the frequency of CD8+CD57+KLRG1+ (p=0.0001) and decreased CD8+CD57+PD-1+ (p<0.05) (**Figure 4D**). More details of comparisons, including HIV- groups, are displayed in **Supplementary Table S3**.

Analysis in the follow-up with CT and VGC+cART treatments showed that the frequency of CD8+CD27+ T-cells positive to KLRG1 (**Figure 4E**) and TIM3+ (**Figure 4G**) and CD8+CD57+KLRG1+ T-cells (**Figure 4H**) was unchanged. The frequency of CD8+CD27+PD-1+ T-cells at baseline was higher in VGC+cART than in CT (p<0.05). However, it decreased significantly at W_4_ (p < 0.05) and increased at W_12_ (p < 0.05); in contrast, treatment with CT induced an increase at W_4_ compared to W_0_ but decreased again at W_12_ (**Figure 4F**). VGC+cART induced an increase of CD8+CD57+PD-1+ at W_12_ compared to W_0_ (p<0.001), and from W_4_ onwards, VGC+cART showed a higher frequency than CT (**Figure 4I**). At baseline, VGC+cART showed a higher frequency of CD8+CD57+TIM-3+ than CT (p<0.05), and a decrease at W_12_ (p<0.05) to similar levels as CT (**Figure 4J**). These results suggest that T-cells with a senescent-like T-cell profile are maintained in DKS/HIV patients characterized by KLRG1 expression before either treatment, and PD-1 expression across follow-up.

### 3.5 Unaltered plasma levels of cytokines in DKS/HIV patients show a relationship at baseline

**Figure 5:**
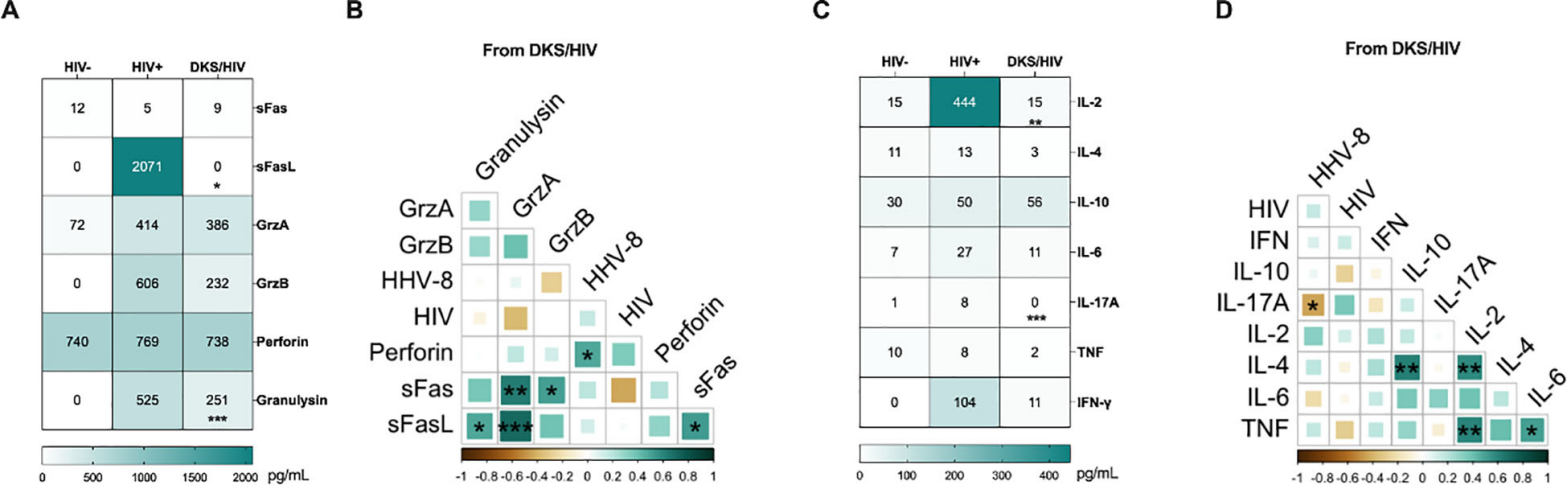
Plasma levels of soluble immune mediators in DKS/HIV patients. **(A)** Heatmap of the plasma levels of cytotoxic molecules between the study groups. **(B)** Spearman correlation between cytotoxic molecules and VL to HIV-1 and HHV-8 in DKS/HIV patients at diagnosis. **(C)** Heatmap of the plasma levels of cytokines between the study groups. **(D)** Spearman correlation between cytokines and VL to HIV-1 and HHV-8 in DKS/HIV patients at diagnosis. Data are represented as median and IQR values. Statistical comparisons were performed by the Kruskal-Wallis’ test; * p < 0.05, ** p < 0.01, *** p < 0.001, **** p < 0.0001. VL= viral load.

Considering that CD8+ T-cells with an exhausted or senescent phenotype are increased in DKS/HIV, plasma levels of cytokines and cytotoxic molecules were analyzed. Data showed that at W_0_, DKS/HIV patients had lower levels of diverse cytotoxic molecules than HIV+ subjects. However, those values were more like HIV-negative individuals, suggesting that HIV infection increases these levels, but coinfection with HHV-8 decreases them (**Figure 5A**). More details of the comparisons, including the HIV group, are provided in **Supplementary Table S5**. The correlation analysis showed positive correlations between perforin with HHV-8 VL (r=0.480, P<0.0308), sFas and Granzyme A (r=0.64, P<0.0025) and Granzyme B (r=0.52, P<0.019), sFaL with granulysin (r=0.487, P<0.029), Granzyme A (r=0.70, P<0.00055) and sFas (r=0.53, P<0.0163) (**Figure 5B**).

Across the follow-up, the VGC+cART scheme induced only an increase in granulysin at W_12_ compared to W_0_ (p < 0.0034); the other cytotoxic levels remained unchanged (**Supplementary Figure S6**).

Regarding cytokine levels, DKS/HIV showed lower levels than HIV+, but they were more like HIV- (**Figure 5C**, **Supplementary Table S4**). A negative correlation was observed between IL-17A and HHV-8 VL (r=-0.44, P<0.049), and positive correlations between IL-4 with IL-10 (r=0.62, P<0.0035) and IL-2 (r = 0.58, P<0.0070), TNF and IL-2 (r=0.606, P<0.0046) and IL-6 (r = 0.52, P<0.019) (**Figure 5D**). Trough the follow-up, the CT scheme increased IL-2, and IL-17A levels at W_12_ compared to W_0_ (IL-2, p<0.014) and W_4_ (IL-17A, p<0.014). Others, such as TNF, IL-6, IL-10, IFN-γ, and IL-4, remained unchanged. (**Supplementary Figure S7**).

### 3.6 High plasma Gal-9 and sTIM-3 levels in DKS/HIV patients show a relationship at baseline and across follow-up

**Figure 6:**
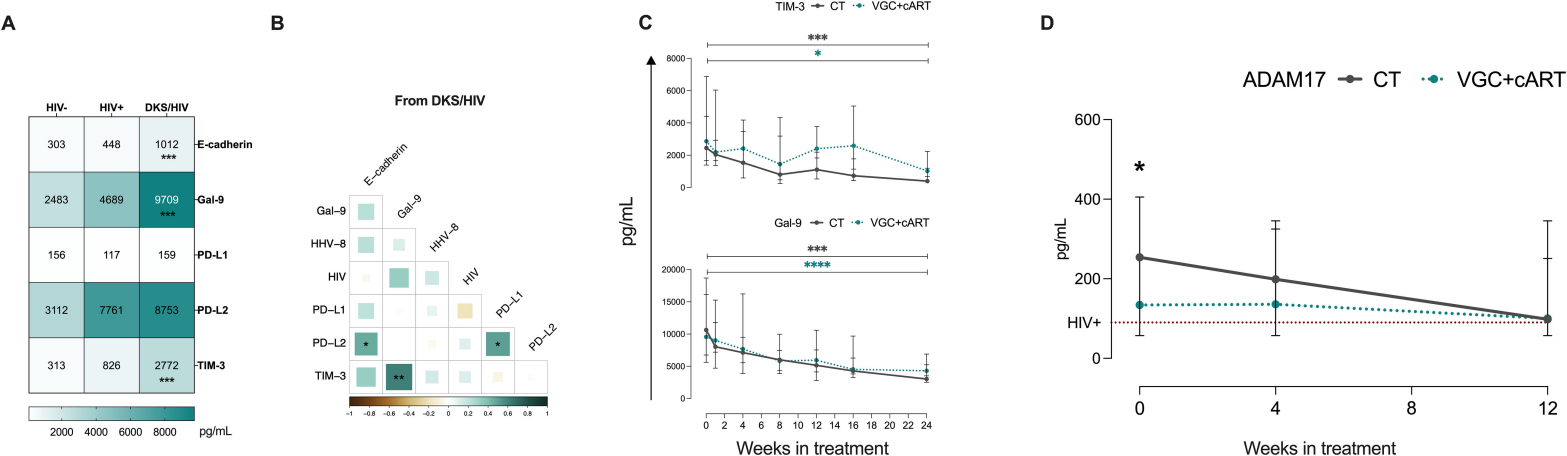
High Gal-9/TIM-3 levels are associated with the diagnostic and follow-up in DKS/HIV patients. **(A)** Heatmap of the plasma levels of E-cadherin, Gal-9, PD-L1, PD-L2, and TIM-3 between the study groups. **(B)** Spearman correlation matrix between E-cadherin, Gal-9, PD-L1, PD-L2, and TIM-3 and VL to HIV-1 and HHV-8 in DKS/HIV patients at diagnosis. **(C)** Gal-9 and TIM-3 soluble levels were assessed by ELISA in the study groups during the clinical follow-up. **(D)** ADAM17 soluble levels were assessed by ELISA in the study groups during the clinical follow-up. Data are represented as median and IQR values. Statistical comparisons were performed by the Kruskal-Wallis’ test; * p < 0.05, ** p < 0.01, *** p < 0.001, **** p < 0.0001. VL= viral load.

The influence of soluble ligands on KLRG1 (E-cadherin), PD-1 (PD-L1/PD-L2), and TIM-3 (Gal-9) could have clinical implications. A previous report with the same cohort of patients demonstrated that PD-L1 levels, but not E-cadherin, were decreased in DKS/HIV patients across follow-up (15). Thus, soluble E-cadherin, Gal-9, PD-L1, PD-L2, and TIM-3 were evaluated. Data showed that plasma levels of Gal-9 and TIM-3 are higher in DKS/HIV patients than in HIV+ subjects (p=0.0001). In contrast, E-cadherin and PD-L2 levels differed only compared to HIV-negative individuals (**Figure 6A**, **Supplementary Table S5**), while PD-L1 did not show significant differences. As shown in **Figure 6B**, plasma Gal-9 levels were positively correlated with soluble TIM-3 levels (p=0.005). Both VGC+cART and CT schemes induced decreased TIM-3 levels at W_12_ compared to W_0_ (VGC+cART, p<0.05; CT, p<0.001), and a similar pattern was observed for Gal-9 levels (VGC+cART, p<0.0001; CT, p<0.001) (**Figure 6C**). High levels of soluble TIM-3 may be linked to increased ADAM17 protease activity. Therefore, its soluble levels were measured. Data showed differences between the HIV+ and CT groups at baseline (P = 0.03). Although there was a trend toward decreasing plasma levels, no significant differences were observed between either VGC+cART or CT treatment schemes at W_0_ or throughout follow-up (**Figure 6D**).

### 3.7 A protease signature is associated with senescence in plasma DKS/HIV patients

To clarify the influence of CT and VGC+cART treatments on the senescent/exhausted-like status exhibited by T-cells of DKS/HIV patients, we evaluated other proteases, whether associated with senescence or not, using a human protease array kit (containing 35 proteases). (**Supplementary Figure S8A**, **C**). Using eight plasma samples from DKS/HIV patients at W_0_ and W_12_, the data indicated that the plasma protease spectrum changed significantly with the therapy schemes (**Supplementary Figure S8B**, **D**).

**Figure 7:**
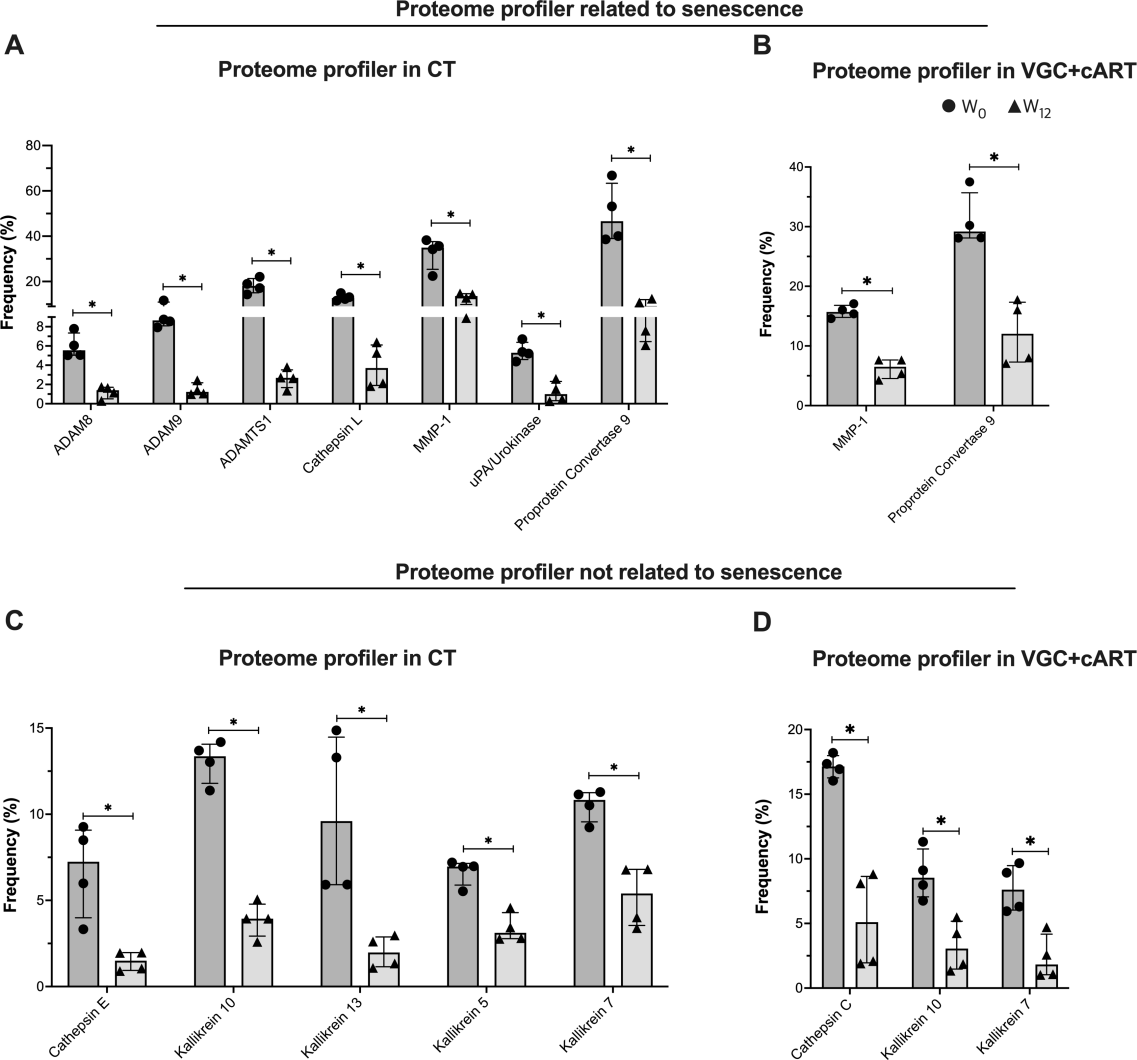
Treatment downregulates the plasma protease profile in DKS/HIV patients. According to the manufacturer’s instructions, plasma proteases were detected using human protease array kits. Frequency of proteome profiles that showed statistically significant changes in DKS/HIV patients with **(A)** CT and **(B)** VGC+cART at diagnosis (•) and after twelve weeks of treatment (δ). The frequency of other plasma protease proteome profiles showed changes that were statistically significant in DKS/HIV patients with **(C)** CT and **(D)** VGC+cART at diagnosis (•) and after twelve weeks of either treatment (δ). Data are represented as median and IQR values. Statistical comparisons were performed by the Mann–Whitney U test (black asterisk); * p < 0.05.

Comparing W_0_ and W_12_, CT induced a decrease in seven proteases related to senescence: ADAM8, ADAM9, ADAMTS1, Cathepsin L, MMP-1, uPA/Urokinase, and proprotein convertase 9 (all with similar p values: 0.0286) (**Figure 7A**). In contrast, VGC+cART induced only the decrease of two: MMP-1 and proprotein convertase 9 (p=0.0286 for both proteases) (**Figure 7B**).

Twelve other proteases unrelated to senescence were also evaluated, and some of them were modified in response to the therapy schemes (**Supplementary Figures S8B, D**). Comparing W_0_ and W_12_, CT decreased five proteases: Cathepsin E, Kallikrein 10, Kallikrein 13, Kallikrein 5, and Kallikrein 7 (all with similar p values: 0.0286) (**Figure 7C**). VGC+cART decreased three proteases: Cathepsin C, Kallikrein 10, and Kallikrein 7 (p=0.0286 for all proteases) (**Figure 7D**). This result indicates that both treatment schemes decrease proteases associated with senescence. However, CT induced a stronger downregulation of the profile, while VGC+cART affected a lower number of proteases.

## 4 Discussion

The prevalence of KS has steeply decreased since highly effective cART became available. Still, KS patients experience severe disease progression, including severe IRIS, characterized by high HHV-8 viral loads and increased mortality within the first weeks of cART initiation in countries that have access to cART. This is particularly true where early HIV diagnosis is still delayed. PLWH often exhibit immune dysfunction that resembles the immune aging process (immunosenescence). The immunosenescence phenotypes in T-cells have been associated with KS. We hypothesized that T-cell aging markers could be prominent in DKS/HIV patients, even during effective therapy and clinical follow-up. Here, our findings suggest that DKS/HIV patients exhibit increased markers of T-cell immunosenescence, enhanced glycolytic activity, and a microenvironment that is likely to promote T-cell senescence.

Glut1 is the principal glucose transporter on T-cells, and its expression is associated with immune activation and HIV-1 replication (7, 19). We found that untreated DKS/HIV patients had significantly elevated frequencies of CD4+ GLUT1+ and CD8+ GLUT1+ T-cells compared to HIV-negative individuals, a pattern similar to that previously observed in HIV+ treatment-naïve patients. Although there is a decrease with suppressive cART, these levels are not completely normalized compared to those in uninfected individuals (19).

Previous studies have shown that GLUT1 remains elevated in CD8^+^ T cells even after cART, suggesting that chronic HIV-1 infection may result in abnormal glucose metabolism (21). We speculate that, in DKS/HIV patients, HHV-8 could synergize with HIV to enhance GLUT1 expression. We did not examine the cause of differential GLUT1 expression in T-cells; however, data have shown that HHV-8 can induce the expression of cellular hypoxia-inducible factors (HIFs), particularly HIF-1α, which upregulates GLUT1 transcription and may promote sustained glucose uptake and metabolic activation, even under cART (22–24).

Unexpectedly, we observed that the frequency of GLUT1+ T-cells did not return to normal levels after treatment, even doubling by week 12 of follow-up, despite viral load suppression and CD4+ T-cell recovery. Although reports indicated that chemotherapy affects GLUT1 expression levels (25), our analysis ruled out this possibility in this study. GLUT1 upregulation is associated with T-cell exhaustion, particularly under hypoxic conditions. Previous studies have also demonstrated a correlation between exhausted CD8+ T-cells and elevated GLUT1 expression, enhanced glucose metabolism, and an immunosuppressive microenvironment (26).

We hypothesized that the high frequency of GLUT1+ T-cells might be associated with the coinfection HHV-8/HIV, and that even after treatment, it could induce immunosenescence. To explore this further, we evaluated the presence of an immunosenescence-like phenotype on T-cells (CD27- CD57+). We found that DKS/HIV patients had increased frequencies of these senescent phenotypes compared to both HIV- and HIV+ individuals without KS. This data expands on our previous observations of sustained T-cell activation in DKS/HIV patients over time (18), adding evidence for a shift toward senescence.

Moreover, CD4+CD57+ T-cells were associated with HIV viral load, while CD8+CD57+ T-cells correlated with HHV-8 viral load and plasma IL-10 levels. IL-10 is known to suppress CD8+ T-cell proliferation, cytokine production (like IFN-γ), and cytotoxicity, potentially preventing excessive immune responses (27). These findings suggest that viral co-infection and associated immune modulation may accelerate immunosenescence and weaken anti-tumor responses. This interpretation could be in consonance with the higher susceptibility to classic KS observed in elderly patients, supporting the idea that immune aging plays a key role in KS pathogenesis.

Another question in our study was whether the observed immunosenescent T-cell phenotypes are modulated by the two therapeutic strategies (CT or VGC+cART), as previously seen with NK and NKT cell modulation in DKS/HIV patients (15). Our results suggest partial normalization of senescence markers, particularly CD57 expression on CD4+ T cells, following CT. The significant decline observed at week 12 may indicate immune recovery and the generation of new, less differentiated CD4+ T cells.

On the contrary, CD8+CD57+ T cells remained elevated across follow-up, even surpassing CD8+CD27+ T cells. Although these cells are important for immune surveillance, their terminally differentiated state limits their proliferative capacity and responsiveness to new antigens (28). This persistence may indicate reduced flexibility in the immune repertoire of DKS/HIV patients.

We also identified distinct immune checkpoint expression profiles in DKS/HIV patients. Consistent with previous reports, PD-1 expression on T cells was elevated in PLWH, especially in untreated patients (29, 30). Additionally, CD4+ T cells in DKS/HIV patients expressed high levels of KLRG1, a marker of T-cell senescence or terminal differentiation, and impaired cytokine production (31). Promisingly, CD4+KLRG1+ cells decreased across follow-up, suggesting partial restoration of CD4+ T-cell function. Interestingly, VGC+cART treatment induced a senescent phenotype in CD8^+^ T cells, marked by late PD-1 acquisition, an effect consistent with prior findings in valganciclovir-treated patients to control asymptomatic CMV (32).

The role of soluble molecules as serologic markers of immunosenescence is critical in understanding viral infections. Consistent with a previous report (33), our study identified that HIV+ individuals have increased sFasL levels; however, DKS/HIV patients showed a significant reduction in sFasL levels. It has been suggested that an sFas/sFasL imbalance is a mechanism that allows the infection to persist (34). Thus, the affected sFasL levels in DKS/HIV may reflect impaired apoptotic signaling in co-infected individuals, which could potentially contribute to viral persistence and immune evasion. The low plasma levels of granulysin observed in DKS/HIV patients also reinforce the idea that HHV-8 evades immune recognition.

Our data showed that, throughout the follow-up, granulysin was upregulated by VGC+cART treatment. Granulysin levels are known to be inhibited by HIV infection, which interferes with signaling pathways in immune cells (35). Effective viral control by cART or VGC+cART regimens could, indirectly, enhance granulysin levels, thereby restoring signaling pathways and strengthening the immune response. A similar negative regulation has been reported for IL-2 plasma levels, and a reduced frequency of CD8+ T cells producing IL-17 has been associated with persistent immune activation in HIV-1 infection (36, 37).

Soluble ligands of checkpoint receptors may further contribute to the immunosenescent profile. For example, high E-cadherin plasma levels can be considered a marker of cell surface dysfunction, given that HHV-8 disrupts E-cadherin and increases endothelial permeability (38). However, when soluble E-cadherin binds to the extracellular domain of KLRG1, the ITIM tyrosine is phosphorylated, contributing to cellular senescence (31). Similarly, high levels of soluble TIM-3, shed by proteases such as ADAM10 or ADAM17, may impair T-cell function by disrupting IL-2 and IFN-γ (39, 40). The TIM-3/Gal-9 axis favors immunosuppressive pathways; for instance, CD8+ T cells from patients with chronic hepatitis B infection exhibit overexpression of TIM-3, leading to antiviral T cell dysfunction (41). To note, soluble TIM-3 and Gal-9 levels decreased over follow-up, regardless of treatment, in DKS/HIV patients, a phenomenon also reported during immune reconstitution in HIV+ individuals after completing treatment. Therefore, soluble TIM-3 and Gal-9 levels could serve as markers of reconstitution in DKS/HIV individuals.

Senescent cells often adopt a senescence-associated secretory phenotype (SASP), releasing proteases, cytokines, and growth factors (42). In addition to ADAM17 changes, we identified distinct protease profiles in DKS/HIV patients after 12 weeks of therapy. CT-treated patients downregulated a broader set of proteases, including ADAM8, ADAM9, ADAMTS1, cathepsins (E and L), kallikreins (5, 7, 10, and 13), and urokinase-type plasminogen activator (UPA), whereas VGC+cART-treated patients showed regulation of cathepsin-C, kallikreins (7 and 10), MMP-1, and proprotein Convertase 9. Several of these proteases modulate extracellular matrix (ECM) components, which are relevant to KS diagnosis, and HHV-8 degrades ECM proteins (43). The protease profile displayed by DKS/HIV patients likely influences T-cell differentiation and senescence. For example, ADAM8 and MMP-1 are implicated in T-cell maturation and ECM remodeling, while cathepsins, UPA, and PC9 contribute to immune dysfunction and aging (44–48). Interestingly, this protease profile is affected after either treatment, suggesting that the expression of senescent-involved molecules is regulated, which could positively impact patient outcomes.

This study has several limitations. First, the sample size is small, and the follow-up period is short; we need to evaluate senescence patterns after years of adherent ARV therapy. Second, other KS groups, like classic KS or iatrogenic KS, were not included in our study, which limits our ability to generalize findings beyond HIV-associated KS. Third, while we assessed several senescence markers, a more comprehensive immune checkpoint panel could provide additional insights into exhaustion and immune regulation in DKS/HIV.

Our findings provide evidence to support the concept that DKS/HIV coinfection represents a model of accelerated immune aging. Even with virologic suppression and immune recovery, patients retain senescent and dysfunctional T-cell populations. The persistence of markers such as GLUT1, CD57, PD-1, KLRG1, and soluble TIM-3/Gal-9, along with altered protease profiles, suggests a chronic immunosuppressive microenvironment that may impair tumor surveillance and immune resilience. Moreover, GLUT1, PD-1, and TIM-3 showed the potential to be explored as biomarkers to monitor response or therapeutic targets to reverse immunosenescence in future studies.

In conclusion, this study provides evidence that immunosenescence is a key component in the microenvironment generated during DKS in PLWH. Future research should include classic KS cohorts and investigate interventions that target metabolic and checkpoint pathways to improve immune restoration and disease control.

## Data Availability

The original contributions presented in the study are included in the article/**Supplementary Material**. Further inquiries can be directed to the corresponding author/s.
