# Supplementary material for: Disseminated Kaposi sarcoma patients exhibit an expanded population of CD8+CD57+ T cells and an immunosenescence profile
Source: Front Immunol. 2025 Oct 24;16:1625386. doi: 10.3389/fimmu.2025.1625386 (PMC12592047; doi:10.3389/fimmu.2025.1625386)
Supplement: Supplementary file 1 [file Table1.docx]

Supplementary Material

# Supplementary Tables

**Table S1.** Antibodies used for flow cytometry and ELISA.

| **Antibody** | **Conjugate to** | **Clone^1^** | **Company** |
| --- | --- | --- | --- |
| CD3 | APC‐Cy7 | OKT3 | Biolegend |
| CD4 | Pacific blue | OKT4 | Biolegend |
| CD8 | BV510 | SK1 | Biolegend |
| CD27 | PercP-Cy5.5 | MT271 | Biolegend |
| CD57 | PE-Cy7 | HNK-1 | Biolegend |
| KLRG-1 | FITC | 2FI/KLRG1 | Biolegend |
| PD-1 | APC | EH12.2H7 | Biolegend |
| TIM-3 | PE | A18087E | Biolegend |
| Viability | PE TexRed | NA | Biolegend |
| Human CD8/NK Panel | NA | 740267 | Biolegend |
| Human TIM-3 DuoSet ELISA | NA | DY2365 | R&D systems |
| Human Galectin-9 DuoSet ELISA | NA | DY2045 | R&D systems |
| Proteome Profiler Human Protease Array Kit | NA | ARY021B | R&D systems |

^1^ Clone or catalogue number.

| **Table S2. Clinical characteristics of DKS/HIV patients.** | | | | | | | | |
| --- | --- | --- | --- | --- | --- | --- | --- | --- |
| **Age (years)** | **IRIS (%)** | **IRIS (# patients)** | **Opportunistic infections, n (%)** | | **Weeks** | **Viral load** | | **CD4/CD8 ratio** |
|  |  |  |  |  |  | **HIV-1** | **HHV-8** |  |
| 33  (26-41) | 11 (55%) | W_2_ (1)  W_4_ (7)  W_6_ (2)  W_9_ (1) | **Syphilis** | 3 (15%) | **W_0_** | 253 568  (88 528-771 162) | 819  (250-4 214) | 0.07  (0.08-0.14) |
|  |  |  | **Mycobacterium avium complex (MAC)** | 3 (15%) | **W_4_** | 17 706  (325-168 739) | 439  (125-1 934) | 0.24  (0.2-0.4) |
|  |  |  | **Histoplasmosis** | 1  (5%) | **W_8_** | 119  (76-319) | 292  (65-4363) | 0.23  (0.1-0.3) |
|  |  |  | **Penicilliosis** | ND | **W_12_** | 61  (40-166) | 795  (60-5 548) | 0.20  (0.14-0.3) |
|  |  |  | ***Helicobacter pylori*** | 3 (15%) | **W_16_** | 40  (40-71) | 250  (50-1 103) | 0.22  (0.1-0.4) |
|  |  |  | **Hepatitis C virus** | ND | **W_24_** | 40  (40-40) | 40  (40-1 456) | 0.27  (0.3-0.26) |

Data are represented as median with interquartile range (IQR, 25–75). ND: undetectable. HIV, Human immunodeficiency virus 1. DKS/HIV: Disseminated Kaposi Sarcoma/human immunodeficiency virus. W_X_= Number of week when the IRIS episode was developed.

| **Table S3. Frequency of T-cells subsets in the control groups compared to DKS/HIV patients.** | | | | |
| --- | --- | --- | --- | --- |
| **Population**  **(%)** | **HIV‐Negative**  **[A] (n=5)** | **HIV+ Asymptomatic [B] (n=10)** | **DKS/HIV**  **[C] (n=20)** | ***p* value** |
| **CD4+GLUT1+** | 2 (1-4) | 13 (3-23) | 8 (3-13) | 0.0203 [A vs C]  0.0431 [A vs B] |
| **CD8+GLUT1+** | 0.5 (0.5-5) | 2 (0-16) | 4 (3-9) | 0.0412 [A vs C] |
| **CD4+CD27+** | 41 (31-49) | 36 (24-53) | 21 (17-36) | 0.0372 [A vs C] |
| KLRG1+ | 5 (4-5) | 1 (0.1-5) | 16 (7-36) | 0.0185 [A vs C] |
| PD-1+ | 3 (1-5) | 13 (7-23) | 1 (0.4-3) | 0.0010 [A vs B] |
| TIM-3+ | 0.4 (0-2) | 0.4 (0-3) | 0.3 (0-4) | ns |
| **CD8+CD27+** | 42 (31-68) | 23 (14-33) | 12 (7-17) | 0.0290 [A vs B]  0.0001 [A vs C] |
| KLRG1+ | 0.1 (0.2-0) | 0.1 (0-0.4) | 0 (0-0) | 0.0012 [A vs C] |
| PD-1+ | 2 (1-4) | 40 (20-44) | 1 (0.5-2) | 0.0010 [A vs B] |
| TIM-3+ | 0.2 (0-1) | 7 (4-36) | 0.2 (0-1) | 0.0010 [A vs B] |
| **CD4+CD57+** | 16 (11-17) | 15 (5-21) | 22 (12-32) | ns |
| KLRG1+ | 4 (1-6) | 3 (0-22) | 34 (26-48) | 0.0001 [A vs C] |
| PD-1+ | 3 (1-5) | 7 (5-21) | 2 (1-5) | 0.0190 [A vs B] |
| TIM-3+ | 1 (0-5) | 2 (0-6) | 0 (0-1) | ns |
| **CD8+CD57+** | 22 (9-26) | 22 (9-38) | 42 (19-52) | 0.0427 [A vs C] |
| KLRG1+ | 1 (0.5-5) | 3 (1-6) | 11 (10-20) | 0.0001 [A vs C] |
| PD-1+ | 3 (1-4) | 15 (5-23) | 1 (0.4-2) | ns |
| TIM-3+ | 0.2 (0-0.4) | 0 (0-0.3) | 0 (0-0.4) | ns |

Data are represented as median with interquartile range (IQR, 25–75). Statistical comparisons were performed using the Mann–Whitney U test, CI 95%. (ns: not‐significant). HIV, Human immunodeficiency virus 1. DKS/HIV: Disseminated Kaposi Sarcoma/human immunodeficiency virus.

| **Table S4. Cytokines plasma levels in the control groups compared to DKS/HIV patients.** | | | | |
| --- | --- | --- | --- | --- |
| **Molecules**  **(pg/mL)** | **HIV‐Negative**  **[A] (n=5)** | **HIV+ Asymptomatic [B] (n=10)** | **DKS/HIV**  **[C] (n=20)** | ***p* value** |
| **sFas** | 12 (2-75) | 5 (2-30) | 9 (3-30) | ns |
| **sFasL** | 0 (0-0) | 2071 (2024-2122) | 0 (0-2073) | 0.0040 [A vs B] |
| **Granzyme A (GzmA)** | 72 (0-391) | 414 (335-464) | 386 (229-791) | 0.0397 [A vs C] |
| **Granzyme B (GzmB)** | 0 (0-0) | 606 (265-678) | 232 (0-732) | 0.0015 [A vs B] |
| **Perforin** | 740 (180-769) | 769 (733-807) | 738 (705-779) | ns |
| **Granulysin** | 0 (0-556) | 525 (386-591) | 251 (0-371) | ns |
| **IL-2** | 15 (0-84) | 444 (151-508) | 15 (0-220) | 0.0066 [A vs B] |
| **IL-4** | 11 (5-53) | 13 (5-22) | 3 (0-34) | ns |
| **IL-10** | 30 (0-31) | 50 (27-69) | 56 (21-107) | 0.0341 [A vs C] |
| **IL-6** | 7 (0-11) | 27 (14-34) | 11 (4-41) | 0.0060 [A vs B] |
| **IL-17A** | 1 (0-6) | 8 (3-13) | 0 (0-1) | 0.0472 [A vs B] |
| **TNF-α** | 10 (0.5-17) | 8 (2-17) | 2 (0-19) | ns |
| **IFN-γ** | 1 (0.2-37) | 104 (75-132) | 11 (8-36) | 0.0001 [A vs B]  0.0012 [A vs C] |

Data are represented as median with interquartile range (IQR, 25–75). Statistical comparisons were performed using the Mann–Whitney U test, CI 95%. (ns: not‐significant). HIV, Human immunodeficiency virus 1. DKS/HIV: Disseminated Kaposi Sarcoma/human immunodeficiency virus.

| **Table S5. Soluble ligands plasma levels in the control groups compared to DKS/HIV patients.** | | | | |
| --- | --- | --- | --- | --- |
| **Molecules**  **(pg/mL)** | **HIV‐Negative Men**  **[A] (n=5)** | **HIV+ Asymptomatic Men [B] (n=10)** | **DKS/HIV**  **[C] (n=20)** | ***p* value** |
| **E-Cadherin** | 303 (231-420) | 448 (273-612) | 1012 (756-1732) | 0.0001 [A vs C] |
| **Gal-9** | 2483 (1842-019) | 4689 (2442-6917) | 9709 (6888-15014) | 0.0001 [A vs C]  0.0274 [A vs B] |
| **PD-L1** | 156 (156-160) | 117 (78-455) | 156 (156-308) | ns |
| **PD-L2** | 3112 (2159-4412) | 7761 (4567-9183) | 8753 (6619-10327) | 0.0001 [A vs C] |
| **TIM-3** | 313 (313-414) | 826 (780-943) | 2772 (1647-4890) | 0.0001 [A vs C]  0.0001 [A vs B] |

Data are represented as median with interquartile range (IQR, 25–75). Statistical comparisons were performed using the Mann–Whitney U test, CI 95%. (ns: not‐significant). HIV, Human immunodeficiency virus 1. DKS/HIV: Disseminated Kaposi Sarcoma/human immunodeficiency virus.

# Supplementary Figures





**Figure S1. DKS/HIV patients’ follow-up strategy.** The groups of men who have sex with men (MSM) were identified in two control groups (HIV+ and HIV-), and samples were collected only at the time of HIV diagnosis. Follow-up was conducted for 20 patients diagnosed with DKS/HIV coinfection: 10 patients were selected to begin cART treatment, and 10 patients were assigned to start VGC treatment. A comprehensive clinical and immunological follow-up was conducted, and a randomized selection was performed for flow cytometry analysis and measurement of systemic cytokine levels at W_0_, W_4_, and W_12_.

**
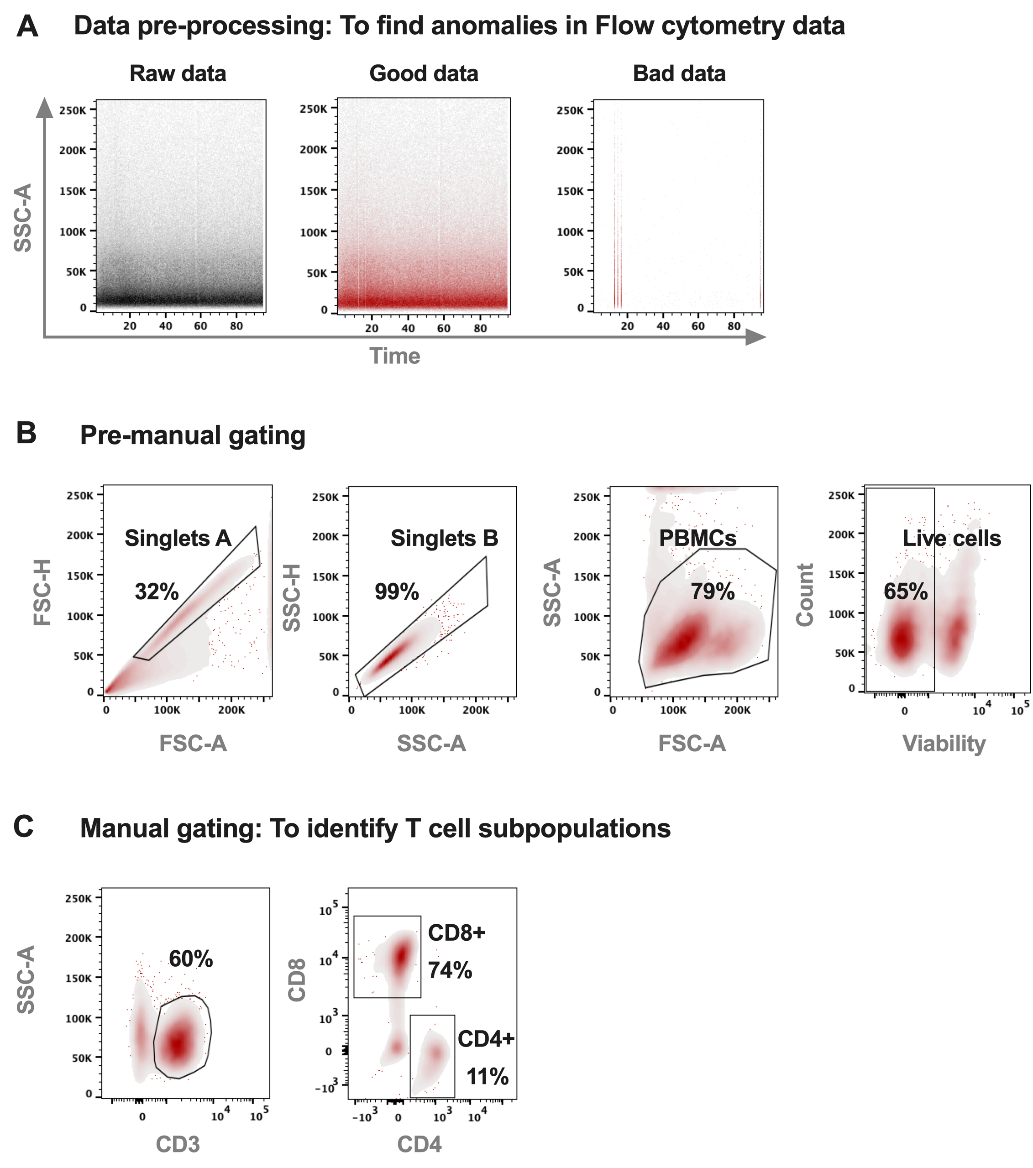
**

**Figure S2. General flow cytometry strategy.** Representative results were obtained in PBMCs from DKS/HIV patients at baseline. The FlowAI analysis was conducted as a quality control measure to detect and remove anomalies from FCS data, examining the flow rate, signal acquisition, and dynamic range in each sample acquired before analysis (**A**). Pre-manual gating was performed on good events identified in FlowAI analysis. Viable cells were selected, and PBMCs were selected through forward scatter (FSC) and side scatter (SSC) dot plot. Finally, single events are identified by forward (FSC-A vs. FSC-H) and side scatter (SSC-H vs. SSC-A) (**B**). As a part of manual gating, T cells were identified based on CD3 expression, and their subsets were selected by CD4 or CD8 expression (**C**).

**
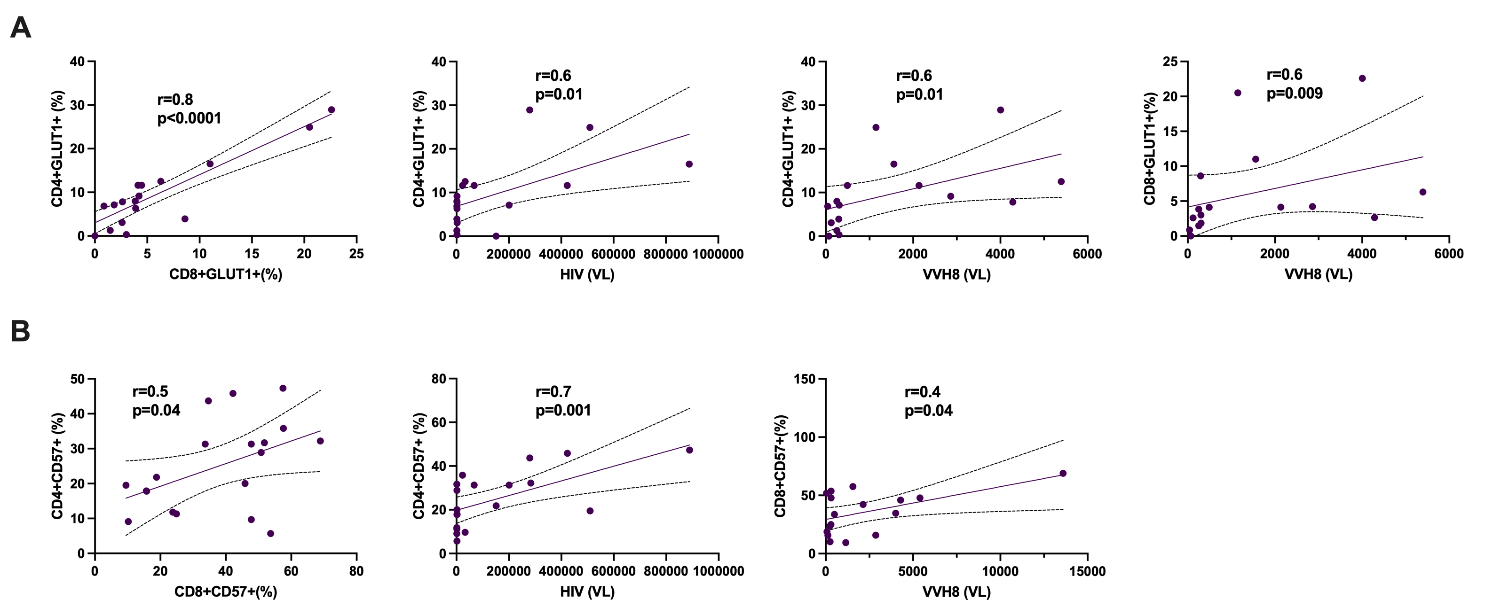
**

**Figure S3. Correlation of evaluated parameters on DKS/HIV patients. (A)** Comparison of the GLUT1+ T cells and CD57+ T cells analysis vs. subsets of T cells, HIV, and HHV8 viral load. (B) Purple lines represent values with 95% confidence intervals. Spearman correlation coefficient (r) is shown for the comparisons.

**
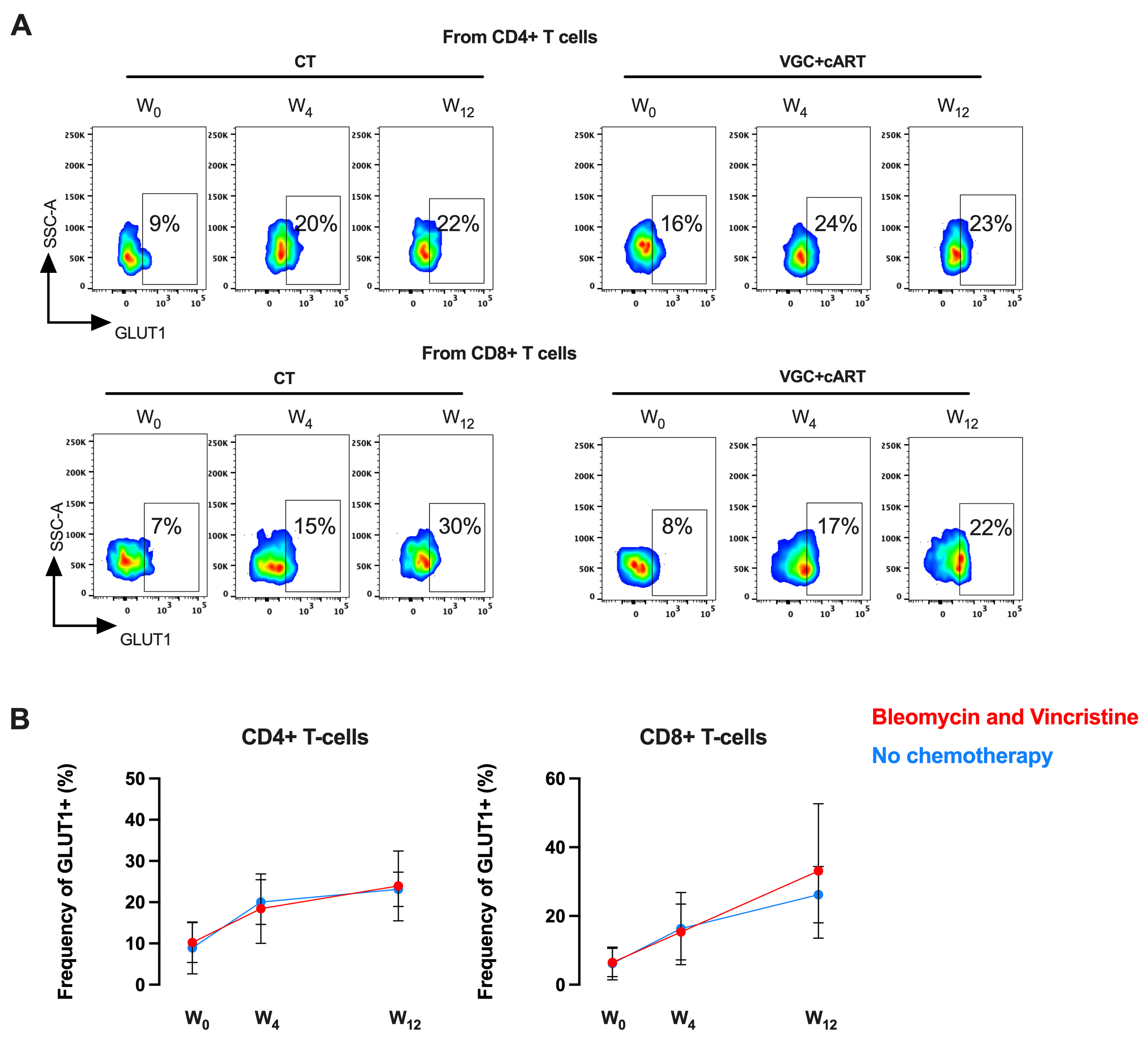
**

**Figure S4. Chemotherapy effects on the GLUT1 expression on T-cells of DKS/HIV patients**. (**A**) Representative flow cytometry plots of GLUT1+ T-cells in the DKS/HIV patient groups across follow-up. (**B**) Frequency of CD4+ GLUT1+ and CD8+ GLUT1+ T-cells in the DKS/HIV patient groups with or without chemotherapy across follow-up (n=10 per group). Data is presented as median and IQR values. Statistical comparisons were performed to compare two groups using the Mann–Whitney U test or through follow-up using the Kruskal-Wallis test.

**
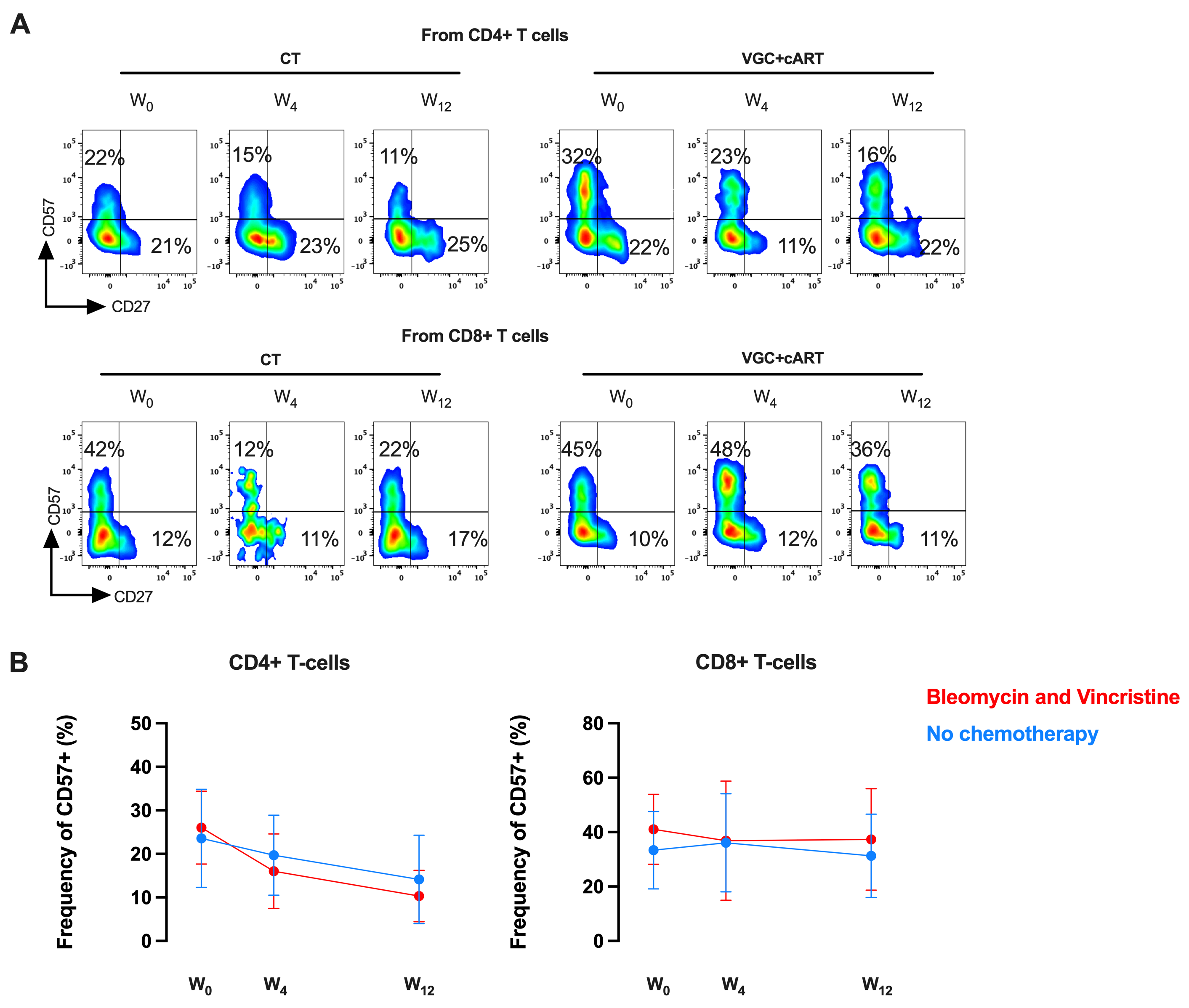
**

**Figure S5. Chemotherapy effects on the CD57+ phenotype acquisition on T-cells of DKS/HIV patients.** (**A**) Representative flow cytometry of the expression of CD27 and CD57 within CD4+ and CD8+ T-cells of HIV+ patients throughout the follow-up of DKS/HIV. (**B**) Frequency of CD4+CD57+ and CD8+CD57+ T-cells during the follow-up of DKS/HIV patients with and without chemotherapy (n=10 per group). Data are represented as median and interquartile range (IQR) values. Statistical comparisons were performed to compare two groups using the Mann–Whitney U test or to assess changes over time using the Kruskal-Wallis test.

**
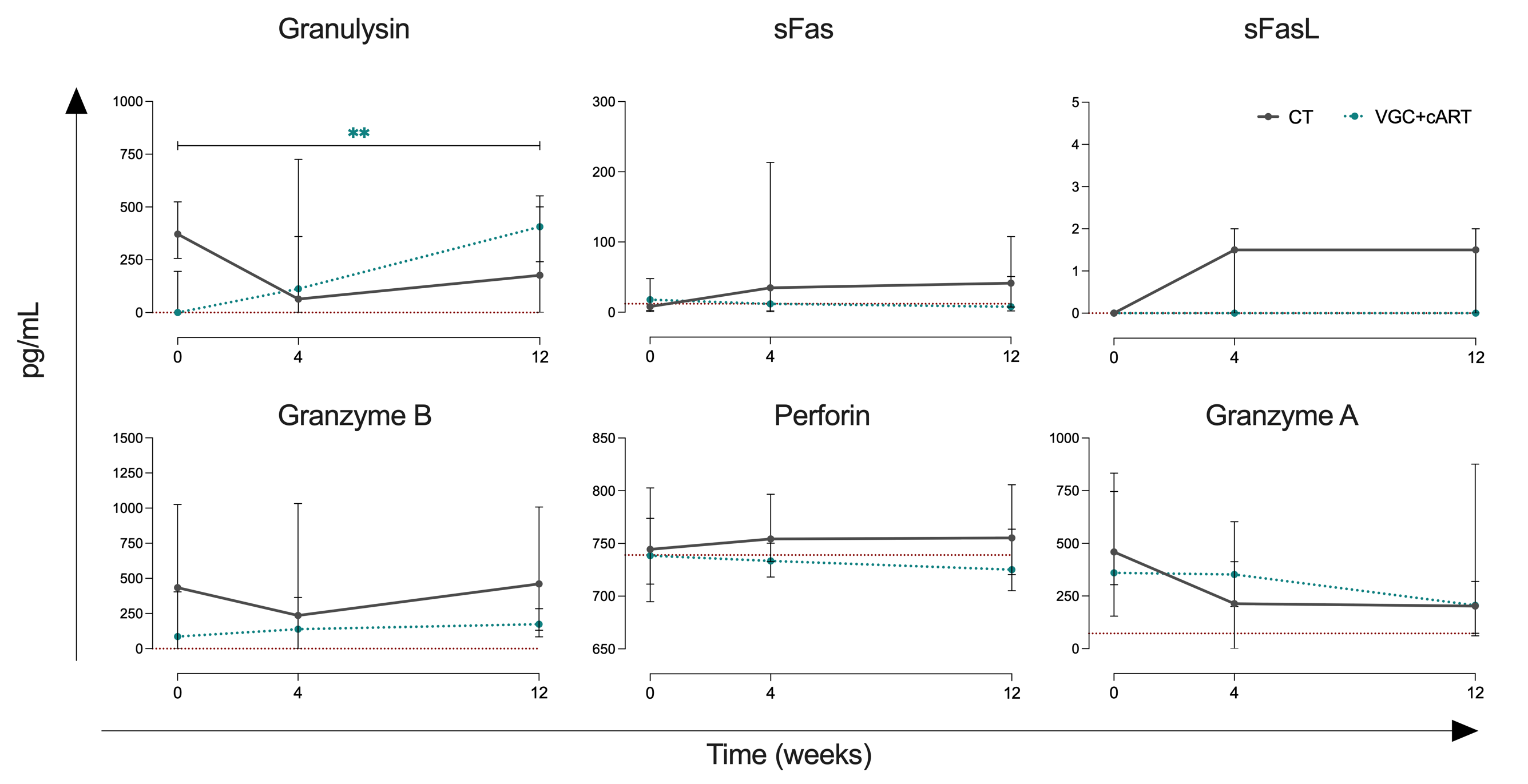
**

**Figure S6. Evaluation of cytotoxic molecules levels across the follow-up in DKS/HIV patients.** Plasmatic levels of cytotoxic molecules in the study groups during the clinical follow-up (n=10 per group). Data are represented as median and IQR values. The red line represents the median value for HIV-negative men. Statistical comparisons were performed by the Kruskal-Wallis test (green for VGC+cART and grey for CT group); * p < 0.05, ** p < 0.01.


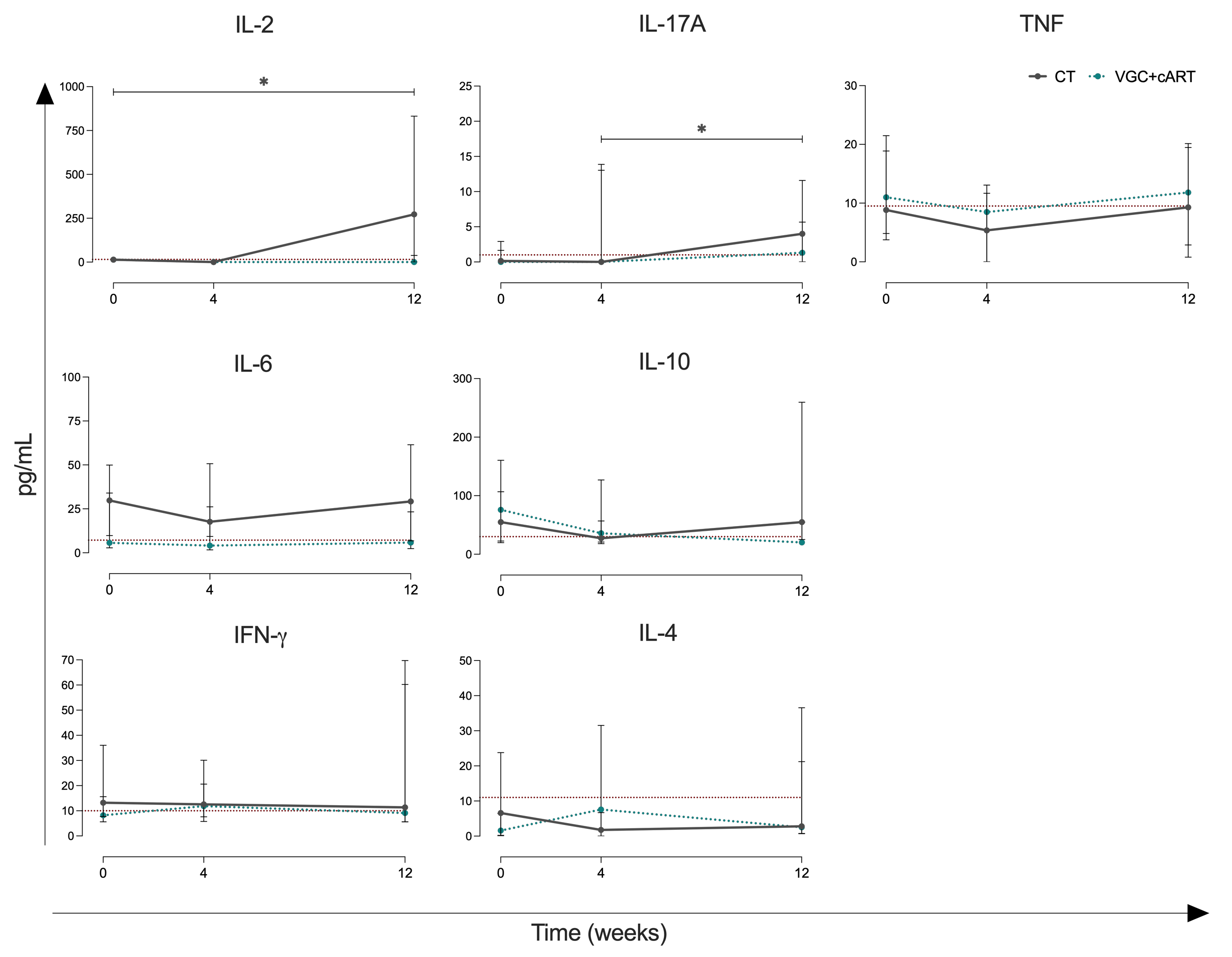


**Figure S7. Evaluation of cytokine levels across the follow-up in DKS/HIV patients.** Plasmatic levels of cytokines in the study groups across the clinical follow-up (n=10 per group). Data are represented as median and IQR values. The red line represents the median value for HIV-negative men. Statistical comparisons were performed by the Kruskal-Wallis test (green for VGC+cART and grey for CT group); * p < 0.05.


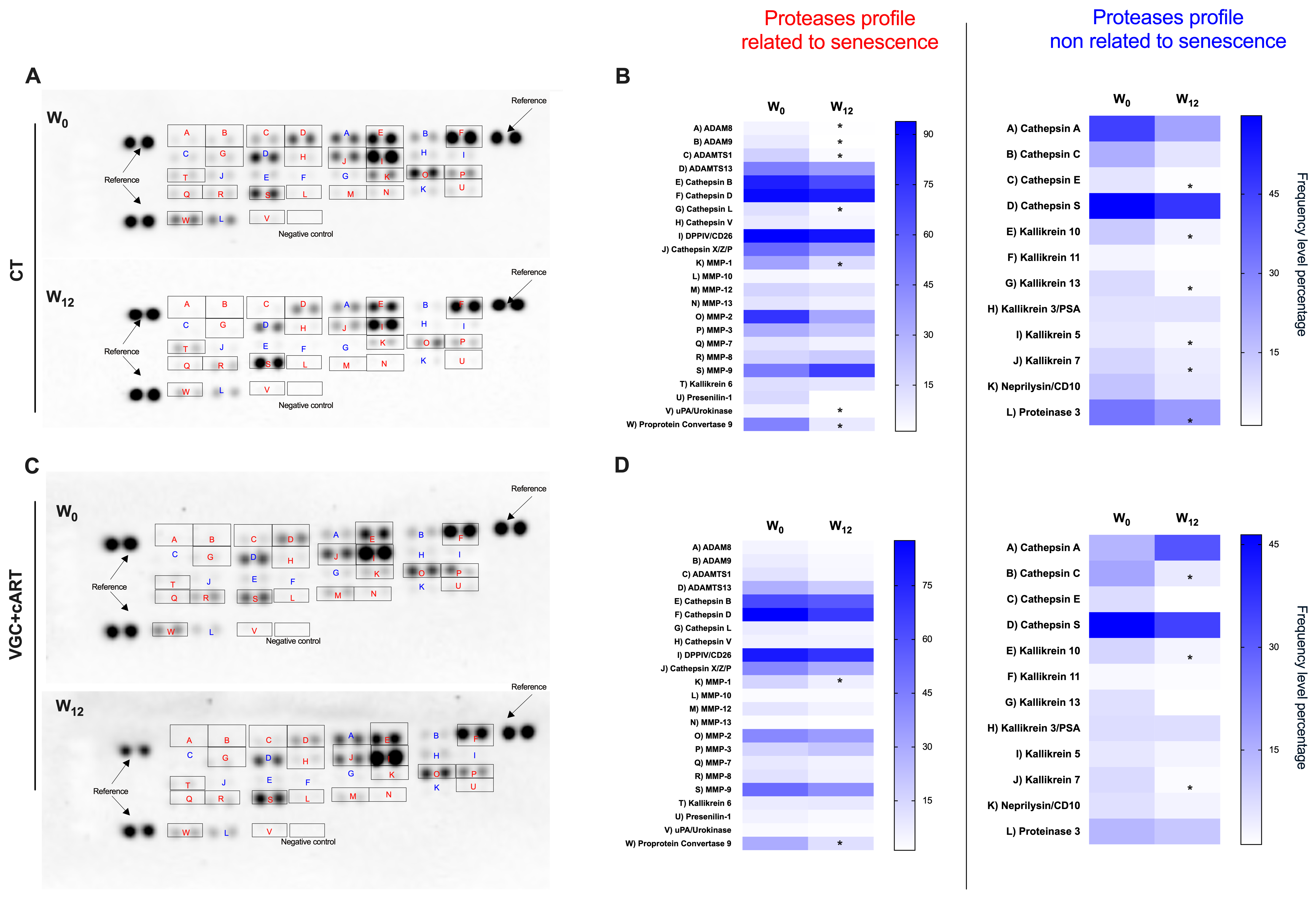


**Figure S8. Analysis of plasma proteases by human protease array kits from DKS/HIV patients.** (**A and B)** Representative pictures of the proteome profiler human protease array in DKS/HIV patients at baseline (W_0_) and after 12 months of treatment (W_12_) compared to the CT group. (Red indicates proteases related to senescence, and blue indicates proteases not related to senescence.) (**C and D**) Quantitative analysis, presented as heat maps of the proteome profile related to or not related to senescence, showed statistically significant changes in DKS/HIV patients under the CT and VGC+cART schemes. Statistical comparisons were performed using the Mann–Whitney U test; * p < 0.05.
